# Autonomous differentiation of transgenic cells requiring no external hormone application: the endogenous gene expression and phytohormone behaviors

**DOI:** 10.3389/fpls.2024.1308417

**Published:** 2024-04-03

**Authors:** Yuka Sato, Mai F. Minamikawa, Berbudi Bintang Pratama, Shohei Koyama, Mikiko Kojima, Yumiko Takebayashi, Hitoshi Sakakibara, Tomoko Igawa

**Affiliations:** ^1^ Plant Cell Technology Laboratory, Graduate School of Horticulture, Chiba University, Matsudo, Japan; ^2^ Institute for Advanced Academic Research (IAAR), Chiba University, Chiba, Japan; ^3^ RIKEN Center for Sustainable Resource Science, Yokohama, Japan; ^4^ Graduate School of Bioagricultural Sciences, Nagoya University, Nagoya, Japan; ^5^ Plant Molecular Science Center, Chiba University, Chiba, Japan; ^6^ Research Center for Space Agriculture and Horticulture, Chiba University, Matsudo, Japan

**Keywords:** autonomous cell differentiation, *BABY BOOM*, *WUSCHEL*, transgenic cells, PGR-free, phytohormone analysis, RNA-seq

## Abstract

The ectopic overexpression of developmental regulator (DR) genes has been reported to improve the transformation in recalcitrant plant species because of the promotion of cellular differentiation during cell culture processes. In other words, the external plant growth regulator (PGR) application during the tissue and cell culture process is still required in cases utilizing DR genes for plant regeneration. Here, the effect of *Arabidopsis BABY BOOM* (*BBM*) and *WUSCHEL* (*WUS*) on the differentiation of tobacco transgenic cells was examined. We found that the *SRDX* fusion to *WUS*, when co-expressed with the *BBM*-*VP16* fusion gene, significantly influenced the induction of autonomous differentiation under PGR-free culture conditions, with similar effects in some other plant species. Furthermore, to understand the endogenous background underlying cell differentiation toward regeneration, phytohormone and RNA-seq analyses were performed using tobacco leaf explants in which transgenic cells were autonomously differentiating. The levels of active auxins, cytokinins, abscisic acid, and inactive gibberellins increased as cell differentiation proceeded toward organogenesis. Gene Ontology terms related to phytohormones and organogenesis were identified as differentially expressed genes, in addition to those related to polysaccharide and nitrate metabolism. The qRT-PCR four selected genes as DEGs supported the RNA-seq data. This differentiation induction system and the reported phytohormone and transcript profiles provide a foundation for the development of PGR-free tissue cultures of various plant species, facilitating future biotechnological breeding.

## 1 Introduction

Plants can regenerate a whole new plant from a single cell through dedifferentiation and redifferentiation, which is an ability named totipotency. Artificial control of totipotency in cell and tissue culture has been used for plant conservation, breeding, and scientific research because it accelerates not only plant propagation, but also the production of genetically modified plants. Cell differentiation is controlled by the external application of plant growth regulators (PGRs), such as auxins and cytokinins, to the culture medium; however, the optimal hormonal conditions for differentiation vary depending on plant species, genotypes, and tissue types. Therefore, establishment of optimal hormonal conditions to induce differentiation is required for every explant type. To date, several genes have been found to affect cell differentiation, such as *ISOPENTENYLTRANSFERASE* (*IPT*) (Akiyoshi et al., 1984; Barry et al., 1984), *SHOOT MERISTEMLESS* (*STM*) (Barton and Poethig, 1993), *LEAFY COTYLEDON1* (*LEC1*) (Lotan et al., 1998), *LEAFY COTYLEDON2* (*LEC2*) (Stone et al., 2001), *GROWTH-REGULATING FACTOR-GRF-INTERACTING FACTOR* (*GRF-GIF*) (Debernardi et al., 2020), *BABY BOOM* (*BBM*) (Boutilier et al., 2002), and *WUSCHEL* (*WUS*) (Laux et al., 1996). Therefore, these genes are also called developmental regulators (DRs).

*BBM* encodes an AP2/ERF transcription factor (Boutilier et al., 2002), which functions as a key regulator of zygotic embryogenesis in *Arabidopsis* (Chen et al., 2022a). Somatic embryo formation from the tissues of regenerated transgenic plants where *Brassica BBM* was overexpressed has been reported in *Arabidopsis*, rapeseed, tobacco, and sweet pepper (Boutilier et al., 2002; Heidmann et al., 2011). Also, during cell culture, the introduction and overexpression of *Brassica BBM* in Chinese white poplar calli induced somatic embryos (Deng et al., 2009).

*WUS* encodes a homeobox transcription factor that mainly localizes to the shoot apical meristem (SAM) to maintain stem cell identity (Laux et al., 1996). *WUS* is also involved in embryogenesis, and its overexpression induces somatic embryogenesis in *Arabidopsis* (Zuo et al., 2002; Ikeda et al., 2009). In addition to its homeodomain, the C-terminal region of the WUS protein contains three transcriptional regulatory domains: an acidic domain, a WUS-box, and an ethylene-responsive element binding factor-associated amphiphilic repression (EAR-like) motif (Ikeda et al., 2009).

Methodology using ectopic expression of these DRs induces somatic embryogenesis and shoot formation in transgenic cells, consequently improving transformation efficiency (Gordon-Kamm et al., 2019; Duan et al., 2022; Lian et al., 2022; Cody et al., 2023). In particular, a combination of *BBM* and *WUS* has been frequently used in biotechnological studies to improve the transformation efficiency of recalcitrant monocot cultivars (Lowe et al., 2016; Mookkan et al., 2017; Lowe et al., 2018; Gordon-Kamm et al., 2019; Suo et al., 2021; Johnson et al., 2023).

However, these previous studies using DRs have not completely omitted the application of PGRs to the tissue or cell culture media for plant regeneration. The main bottleneck in artificial plant cellular differentiation is the difficulty in establishing the appropriate culture conditions, especially PGR application recipe, for each explant material. To overcome the obstacles, a versatile methodology to regulate cellular differentiation using DRs would be useful for biotechnological breedings. In addition, detailed information on the gene expression profiles and physiology regulated by DRs during cell differentiation will be informative for further improvements in plant molecular science and biotechnological breeding. Several studies have reported that phytohormone signaling in regenerated transgenic plants is altered by the ectopic expression of DRs (Li et al., 2019; Chen et al., 2022b; Zhou et al., 2023). However, how DR expression affects endogenous gene expression and physiology in cells undergoing differentiation has not been understood completely.

In this study, we utilized *Arabidopsis* BBM and WUS and modified the expressions and proteins to achieve autonomous differentiation of the transgenic cells. Here, we report that Brassicaceae *Arabidopsis BBM* and *WUS* successfully induced autonomous dedifferentiation and redifferentiation of transgenic cells without the application of PGRs during the culture process in Solanaceae tobacco, petunia, and Asteraceae lettuce. Using the differentiating transgenic tobacco cells obtained with the above system, we performed phytohormone measurements and RNA-seq analyses. We herein provide information on the physiological and gene expression backgrounds that enable autonomous cell dedifferentiation.

## 2 Materials and methods

### 2.1 Plant materials and growth conditions

*Arabidopsis thaliana* (Col-0) seeds were surface-sterilized in sodium hypochlorite solution (1% effective chlorine concentration) for 7–10 minutes, and then germinated on Murashige and Skoog (MS) medium containing 1% sucrose, 0.5 g l^-1^ 2-Morpholinoethanesulfonic acid, monohydrate (MES), and 0.8% agar. Two-week-old seedlings were acclimatized and grown at 22°C under an 8 h light/16 h dark cycle. *In vitro-*grown tobacco (*Nicotiana tabacum* ‘Petit Havana’ SR-1) (Maliga et al., 1973) and petunia (*Petunia x hybrida* ‘White Creepia’) plants were maintained with half-strength MS medium containing 3% sucrose and 0.8% agar at 25°C under continuous fluorescent light. Lettuce (*Lactuca sativa* ‘Cisco’, ‘Watson’, and ‘Berkeley’) (TAKII & Co.,Ltd, Kyoto, Japan) seeds were surface-sterilized in sodium hypochlorite solution (1% effective chlorine concentration) for 20 min, and then grown on half-strength MS medium containing 1% sucrose and 0.8% agar at 22°C under an 8 h light/16 h dark cycle.

### 2.2 Vector construction

The primers used for vector construction are listed in **Supplementary Table 1**. *BBM* (AT5G17430) coding region (CDS) was PCR-amplified using cDNA from *A. thaliana* pistils at 7 day-after flowering and was then cloned into pCR® 8/GW/TOPO (Thermo Fisher Scientific, Inc., Waltham, M.A., USA). The *BBM* CDS was inserted into pGWB2 (Nakagawa et al., 2007) using the LR reaction (Thermo Fisher Scientific, Inc.). The PCR-amplified fragment of *Ipomoea batatas Myb* (*IbMyb*) expression cassette (35Sp:*IbMyb*:NosT), which induces anthocyanin biosynthesis in the transgenic tobacco cells for visible marker selection (Sato et al., 2023), was further inserted into PmeI-SbfI site using the In-Fusion® HD cloning kit (Takara Bio Inc., Shiga, Japan), producing a pGWB_*BBM* binary vector (**Supplementary Figure 1B**).The 35Sp:*ΩBBMVP16*:HSPT region was cloned into an entry vector (pUC_35Sp:*ΩBBMVP16*) and transferred to pGWB1 (Nakagawa et al., 2007) via the LR reaction. Then an *IbMyb* expression cassette was further inserted into PmeI-SbfI site by In-Fusion®, producing a pGWB_*ΩBBMVP16* binary vector (**Supplementary Figure 1C**). The CDS of *WUS* (AT2G17950) was amplified using cDNA from *A. thaliana* flower buds by PCR. The amplicons of *A. thaliana* ribosomal protein subunit 5A (RPS5A) promoter (AT3G11940) and the *WUS* CDS were inserted into a NotI site on pUC_35Sp:*ΩBBMVP16* entry vector through In-Fusion®. The constructed 35Sp:*ΩBBMVP16*:NosT_RPS5Ap:*WUS*:NosT region was amplified, then inserted into the BamHI-SalI site on pKI1.1R vector (Tsutsui and Higashiyama, 2017) through In-Fusion®. Further, the *IbMyb* expression cassette was inserted into a PmeI site by In-Fusion®. The final binary vector was named pKI_*ΩBBMVP16*&*WUS* (**Supplementary Figure 1G**). PCR-amplified fragments of RPS5A promoter, *SRDXWUSm1*:NosT, and 35S promoter were inserted into the HpaI-NruI site on pKI_*ΩBBMVP16*&*WUS* vector through In-Fusion®, producing a binary vector pKI_*ΩBBMVP16*&*SRDXWUSm1* (**Supplementary Figure 1J**). pGWB_*GFP* (**Supplementary Figure 1A**) was obtained by LR reaction of pCR® 8/GW/TOPO_*eGFP* and pGWB2 (Nakagawa et al., 2007). The PCR-amplified 35Sp:*BBM*:NosT region of pGWB_*BBM* (**Supplementary Figure 1B**) was inserted into the XmaI-PsiI sites of pKI_*ΩBBMVP16*&*WUS* (**Supplementary Figure 1G**) through In-Fusion®, producing a binary vector pKI_*BBM*&*WUS* (**Supplementary Figure 1F**). The SmaI-PsiI region was removed from pKI_*ΩBBMVP16*&*WUS* (**Supplementary Figure 1G**), and then self-ligated using Ligation high Ver. 2 (TOYOBO Co. Ltd., Osaka, Japan) to produce the binary vector pKI*_WUS* (**Supplementary Figure 1D**). The SmaI-PsiI regions of pKI_*ΩBBMVP16*&*SRDXWUSm1* (**Supplementary Figure 1J**) were removed and self-ligated to produce the binary vector pKI*_SRDXWUSm1* (**Supplementary Figure 1E**). The *WUS:*NosT:35Sp region was inserted into the PmlI-NruI site on pKI_*ΩBBMVP16*&*SRDXWUSm1* (**Supplementary Figure 1J**) through In-Fusion®, producing a binary vector pKI_*ΩBBMVP16*&*SRDXWUS* (**Supplementary Figure 1I**). The ApaI-PmlI sites of pKI_*ΩBBMVP16*&*SRDXWUSm1* (**Supplementary Figure 1J**) was replaced with the HSPT:RPS5Ap:*WUS* (partial) fragment, producing pKI_*ΩBBMVP16*&*WUSm1* (**Supplementary Figure 1H**). All the series of T-DNA schemes constructed for *Agrobacterium*-mediated gene transfer are shown in **Supplementary Figure 1**. *BBM* and *WUS* expression was controlled by the cauliflower mosaic virus 35S and *A. thaliana* RPS5A promoters, respectively. The expression and functions of *BBM* and *WUS* were examined. For *BBM*, the 5’-leader sequence of tobacco mosaic virus (translational enhancer Ω) (Gallie, 2002) was inserted upstream of the CDS, and a translational activator domain of VP16 from herpes simplex virus (Triezenberg et al., 1988) was fused to the C-terminal (*ΩBBMVP16*, **Supplementary Figure 1C**). For *WUS*, two amino acid mutations were inserted into the WUS-box (WUSm1) and an artificial strong repression domain, Superman Repression Domain X (SRDX) (Hiratsu et al., 2003), was fused to the N-terminal end of WUSm1 (Ikeda et al., 2009) (*SRDXWUSm1*, **Supplementary Figure 1E**).

### 2.3 *Agrobacterium*-mediated gene transfer

The constructed binary vectors were transferred into *Agrobacterium tumefaciens* strain EHA105. *Agrobacterium tumefaciens* containing each binary vector was grown in Lysogeny Broth (LB) liquid medium containing 20 mg l^-1^ rifampicin, 30 mg l^-1^ chloramphenicol, and an appropriate antibiotic for each binary vector (50 mg l^-1^ kanamycin for pGWB, or 100 mg l^-1^ spectinomycin for pKI) at 28°C overnight in a shaking incubator (120 rpm). The *Agrobacterium* solution was diluted with MS liquid medium (OD_600 _= 0.1) containing 3% sucrose and 100 μM acetosyringone (FUJIFILM Wako Pure Chemical Corporation, Osaka, Japan). *In vitro*-grown tobacco leaves were cut into leaf explants (5.5-mm diameter) using a cork borer (NONAKARIKAKI Co., Ltd, Tokyo, Japan). For infection, 10–20 leaf explants were soaked in *Agrobacterium* solution for 1 min, then co-cultured on MS medium containing 3% sucrose and 0.8% agar for 3 days in the dark. Afterward, the leaf explants were transferred to an MS selection medium containing 3% sucrose, 0.8% agar, 40 mg l^-1^ hygromycin B, and 25 mg l^-1^ meropenem (Sumitomo Dainippon Pharma Co., Ltd., Tokyo, Japan), and subcultured to a new MS selection medium every two weeks. Two and four weeks after *Agrobacterium* infection (2WAI and 4WAI), the cell differentiation phenotypes were evaluated by counting the number of calli and shoots originating from each leaf explant culture. Three independent experiments were conducted to evaluate each binary vector.

*Agrobacterium*-mediated transformations for lettuce and petunia were carried out similarly to tobacco with minor modifications. Leaf discs (5.0-mm diameter) were infected, and a half-strength MS medium was used for *Agrobacterium* inoculation and co-culture. After co-culture, the leaf discs were cultured without selection pressure of hygromycin for a few weeks, followed by subculturing to a new MS selection medium containing 10−40 mg l^-1^ hygromycin B, 25 mg l^-1^ meropenem, 3% sucrose, and 0.8% agar. Shoots that emerged from the leaf explants were detached and transferred to the MS selection medium. Each grown and rooted plant were maintained in the culture vessels with MS selection medium.

### 2.4 Genomic PCR analysis

Genomic DNA was isolated from tobacco calli and shoots originating from infected leaf explants. For petunia and lettuce, small leaves derived from the shoots showing hygromycin resistance were used for genomic PCR. The calli and shoots were homogenized in DNA extraction buffer (containing 20 mM Tris-HCl (pH 8.0), 10 mM EDTA (pH 8.0), and 10% SDS) and then centrifuged. After a phenol/chloroform treatment and ethanol precipitation, the obtained pellets were dissolved in water. Primers for *IbMyb*, *Arabidopsis BBM* and *WUS*, and *Virulence D* (*VirD*, for pTiBo542) detection (**Supplementary Table 1**) were used for PCR.

### 2.5 Real-time quantitative RT-PCR (qRT-PCR)

Total RNA was isolated using ISOSPIN Plant RNA (NIPPON GENE Co., Ltd., Tokyo, Japan). The cDNA was synthesized from 200 ng of total RNA using ReverTra Ace® (TOYOBO Co., Ltd). qRT-PCR was performed using KOD SYBR qPCR Mix (TOYOBO Co., Ltd) and Applied Biosystems StepOnePlus Real Time PCR System (Thermo Fisher Scientific, Inc.), using Comparative C_T_ (ΔΔC_T_) method. For all qRT-PCR analyses, *N. tabacum Elongation Factor 1-alpha* (*EF1α*, AF120093) was used as the reference gene (Schmidt and Delaney, 2010). The primers used in each qRT-PCR analysis were listed in **Supplementary Table 1**. All primers that amplify *WUS* specifically do not distinguish *WUS*, *SRDXWUS*, and *SRDXWUSm1*.

### 2.6 RT-PCR analysis

Independent tobacco calli and shoots originating from the infected leaf explants were sampled as putative transgenic individuals. Total RNA was isolated using ISOSPIN Plant RNA. The cDNA was synthesized from 200 ng of total RNA using ReverTra Ace®. RT-PCR was performed using the primers listed in **Supplementary Table 1**.

### 2.7 Sample preparation for phytohormone-quantitative analysis and RNA-seq

**Figure 1:**
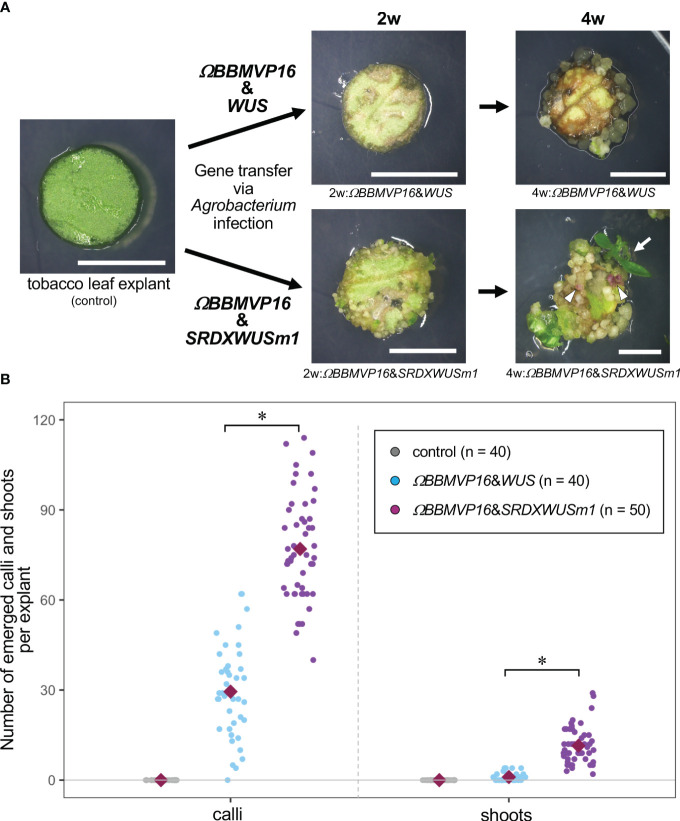
Autonomous differentiation of tobacco leaf cells with introduced *Arabidopsis BABY BOOM* (*BBM*) and *WUSCHEL* (*WUS*) genes. **(A)** The phenotypes of tobacco leaf explant cultures with introduced *ΩBBMVP16*&*WUS* (upper photos) and *ΩBBMVP16*&*SRDXWUSm1* (lower photos) via *Agrobacterium* infection. Photos at two weeks (2w) and four weeks (4w) after *Agrobacterium* infection are shown, respectively. The left image is an explant used for infection (Φ 5.5 mm). A differentiated shoot from the leaf explant cells with introduced *ΩBBMVP16*&*SRDXWUSm1* is marked with a white arrow. White triangles represent anthocyanin pigmentation rarely observed in calli. Bars = 5 mm. **(B)** The number of calli and shoots that emerged from a leaf explant 4 weeks after the infection. Three biological replicates of *Agrobacterium* infection were carried out, and the data is indicated together. Red, green, and blue dot-plots represent the number of calli (left plots) and shoots (right plots) that originated from a non-infected (control) leaf explant or one with introduced *ΩBBMVP16*&*WUS* and *ΩBBMVP16*&*SRDXWUSm1*, respectively. Each violet diamond represents the mean. Asterisks indicate significant differences (*p* < 0.01) between *ΩBBMVP16*&*WUS* and *ΩBBMVP16*&*SRDXWUSm1*, detected by Welch’s t-test.

Five groups were used for phytohormone-quantitative analysis and RNA-seq: non-infected leaf explants (control), leaf cell cultures at 2 and 4WAI with *ΩBBMVP16*&*WUS* (2w:*ΩBBMVP16*&*WUS* and 4w:*ΩBBMVP16*&*WUS*), and leaf cell cultures at 2 and 4WAI with *ΩBBMVP16*&*SRDXWUSm1* (2w:*ΩBBMVP16*&*SRDXWUSm1* and 4w:*ΩBBMVP16*&*SRDXWUSm1*) (**Figure 1A**). Each *Agrobacterium* infection was carried out three or four times as a biological replicate. Five to ten leaf explants were collected.

For phytohormone-quantitative analysis, the collected samples were ground in liquid nitrogen with mortar and pestle and kept at −80°C until the analysis (following section 3.8). For total RNA extraction, the samples were soaked in RNA Save (Cosmo Bio Co., Ltd., Tokyo, Japan) and kept at −80°C until the experiments (following section 3.9).

### 2.8 Phytohormone-quantitative analysis

Phytohormone profiles in the differentiating leaf cultures were analyzed by a highly sensitive and high-through method that allows a single run using ‘MS-probe’ (chemical derivatization) and liquid chromatography-tandem mass spectrometry (Kojima et al., 2009). Phytohormone extraction and semi-purification from ground and frozen tissues (100 mg) were performed as described previously (Kojima et al., 2009; Kojima and Sakakibara, 2012). Contents of cytokinins were quantified with ultra-performance liquid chromatography (UPLC)-electrospray interface (ESI) tandem quadrupole mass spectrometer (qMS/MS) (ACQUITY UPLC™ System/Xevo-TQS) (Waters Corp., Milford, M.A., USA). Abscisic acid (ABA), indole-3-acetic acid (IAA), indole-3-acetyl-L-aspartic acid (IAAsp), gibberellins (GAs), jasmonic acid (JA), jasmonyl-isoleucine (JA-Ile) and salicylic acid (SA) were quantified with ultra-high performance liquid chromatography (UHPLC) electrospray interface (ESI) quadrupole-orbitrap mass spectrometer (UHPLC/Q-Exactive™) (Thermo Fisher Scientific, Inc.) (Shinozaki et al., 2015). The procedures were performed by RIKEN CSRS (Kanagawa, Japan).

### 2.9 RNA-seq analysis

Total RNA was isolated from each sample using ISOSPIN Plant RNA. An RNA-seq library was prepared using the Lasy-Seq ver. 1.1 protocol (Kamitani et al., 2019) using 120 ng of total RNA. Each library was sequenced in triplicates using the single-end mode of the Illumina HiSeqX platform (Illumina Inc., San Diego, C.A., USA). Library preparation and sequencing were performed by Clockmics Inc. (Osaka, Japan).

The raw RNA-seq reads were pre-processed in Trimmomatic ver. 0.39 (Bolger et al., 2014) using default parameters, and the trimmed reads were aligned to the reference tobacco TN90 genome (Sierro et al., 2014) using HISAT2 ver. 2.2.1 (Kim et al., 2015). Gene expression levels were quantified using StringTie ver. 2.2.1 (Pertea et al., 2015). Principal component analysis (PCA) was performed using the graphical user interface for the R package TCC (TCC-GUI ver. 1.0 pipeline) (Su et al., 2019) using the expression data. Differentially expressed genes (DEGs) between the two groups were detected using the TCC-GUI pipeline with a false discovery rate (FDR) of 0.05, and between the three groups using the method of combining the R packages baySeq ver. 2.24.0 and TCC ver. 1.30.0 (Osabe et al., 2019) with FDR of 0.05. The expression levels of the DEGs were visualized using the R package heatmap3 ver. 1.1.9 (Zhao et al., 2014) using read count data normalized by the R package TCC. Gene Ontology (GO) enrichment analysis of the DEGs was performed using the R package clusterProfiler ver. 3.18.1 (Yu et al., 2012). GO term networks were also visualized using the same R package. DEGs were annotated from the NCBI database (https://www.ncbi.nlm.nih.gov/). Orthologous genes of the DEGs in *A. thaliana* were identified using OrthoDB v11 (https://www.orthodb.org/), and annotations of the orthologous genes were obtained from the TAIR database (https://www.arabidopsis.org/). Commonly upregulated DEGs in two–and three–group comparisons were visualized using the R package VennDiagram ver. 1.7.1 (Chen and Boutros, 2011). The raw RNA-seq data was deposited at the NCBI Sequence Read Archive (https://www.ncbi.nlm.nih.gov) with an accession number PRJNA1049661.

## 3 Results

### 3.1 Effects of *Arabidopsis BBM* and *WUS* genes introduced into tobacco, petunia, and lettuce leaf cells

First, we tested the gene expression scheme using *Arabidopsis BBM* and *WUS* as DRs, which enabled autonomous cell differentiation of transgenic tobacco cells in *Agrobacterium*-infected leaf explants during the culture process.

Leaf explants from the experimental controls, non-infected explants, and explants infected with pGWB_35Sp:*GFP*, did not show any reaction to cell differentiation, and were entirely browned by 4WAI (**Supplementary Figure 1A**). The results indicated that tobacco leaf cells could not differentiate or survive on a medium without PGRs, and the stress of *Agrobacterium* infection and gene transfer did not have different effects.

The *BBM* and *ΩBBMVP16*-introduced leaf cells also died by 4WAI (**Supplementary Figures 1B, C**). A previous study reported that overexpression of *SRDXWUSm1* enhanced adventitious shoot formation in *Arabidopsis* (Ikeda et al., 2020). However, *WUS* and *SRDXWUSm1*-introduced leaf cells did not survive (**Supplementary Figures 1D, E**), indicating that the introduction of Arabidopsis *BBM* or *WUS* alone was insufficient to induce tobacco cell differentiation. Next, the combination of *BBM* and *WUS* was examined. Although the introduction of *BBM*&*WUS* was insufficient (**Supplementary Figure 1F**), *ΩBBMVP16*&*WUS-*introduced leaf cells differentiated into calli after 4WAI (**Figure 1A**; **Supplementary Figure 1G**). Furthermore, *ΩBBMVP16*&*SRDXWUSm1*-introduced leaf cells showed a more accelerated differentiation phenotype after 4WAI (**Figure 1A**; **Supplementary Figure 1J**). Unlike the *ΩBBMVP16*&*WUS-*introduced leaf cells, *ΩBBMVP16*&*SRDXWUSm1*-introduced leaf cells differentiated into greenish, organ-like structures, in addition to small calli at 2WAI (**Figure 1A**; **Supplementary Figure 1J**). As of 4WAI, the calli proliferated vigorously, and a few adventitious shoots emerged from *ΩBBMVP16*&*SRDXWUSm1-*introduced leaf cells. Significantly higher numbers of calli and shoots were obtained from leaf explant cultures introduced with *ΩBBMVP16*&*SRDXWUSm1* (**Figure 1B**; **Supplementary Figure 2**). To investigate whether amino acid mutations in WUS-box (WUSm1) or SRDX fusion to the N-terminal of WUS (SRDXWUS) was involved in the accelerated differentiation phenotype of *ΩBBMVP16*&*SRDXWUSm1*-introduced leaf cells, *ΩBBMVP16*&*WUSm1* (**Supplementary Figure 1H**) and *ΩBBMVP16*&*SRDXWUS* (**Supplementary Figure 1I**) were evaluated. While *ΩBBMVP16*&*WUSm1*-introduced leaf cells did not induce any cell differentiation and died by 4WAI (**Supplementary Figure 1H**), *ΩBBMVP16*&*SRDXWUS*-introduced leaf cells differentiated calli and shoots vigorously (**Supplementary Figure 1I**). Compared to *ΩBBMVP16*&*SRDXWUS*, the number of calli that emerged from a leaf explant culture was higher in *ΩBBMVP16*&*SRDXWUSm1*, whereas the number of shoots did not show significant differences between the two constructs (**Supplementary Figure 2**). In the present study, introducing *Arabidopsis ΩBBMVP16*&*WUS*, *ΩBBMVP16*&*SRDXWUS*, and *ΩBBMVP16*&*SRDXWUSm1* succeeded in the autonomous differentiation of tobacco leaf cells without applying PGRs during the tissue culture process.

To confirm the observed phenotypes (**Figure 1**; **Supplementary Figure 1**) were linked to expression of the introduced genes, real-time qRT-PCR was performed for tobacco leaf explants introduced the *ΩBBMVP16*&*WUS* and *ΩBBMVP16*&*SRDXWUSm1* (**Supplementary Figure 3**). As a result, the transcription of *Arabidopsis BBM* and *WUS* was confirmed in the infected leaf explants during the culture by 4WAI. Compared to *WUS* driven by RPS5A promoter, *BBM* driven by 35S promoter showed significantly higher expression levels in all the 2 and 4WAI leaf explants.

We further evaluated whether the autonomously differentiated calli and shoots were the transgenics expressing *BBM* and *WUS*. Genomic PCR analysis was performed using calli and shoots originated from *ΩBBMVP16*&*WUS-* and *ΩBBMVP16*&*SRDXWUSm1*-introduced leaf cells. As a result, the *Arabidopsis BBM* and *WUS* were detected in the callus and shoot lines obtained with *ΩBBMVP16*&*WUS* and *ΩBBMVP16*&*SRDXWUSm1*, but the *VirD* gene from *Agrobacterium* was not, showing that these were transgenic (**Supplementary Figure 4**). *IbMyb*, which has been introduced as a visible marker of anthocyanin pigmentation (Kim et al., 2010; Sato et al., 2023), was also detected; however, anthocyanin pigmentation was hardly observed in the differentiated cells (**Figure 1A**; **Supplementary Figures 1G, I, J**). To further verify that each transgene was transcribed in transgenic calli and shoots, RT-PCR was performed. As a result, all the *IbMyb*, *Arabidopsis BBM*, and *WUS* expressions were detected (**Supplementary Figure 5**). Thus, the downstream anthocyanin biosynthesis pathway induced by IbMyb may have been hampered in cells expressing *BBM* and *WUS*.

Furthermore, we tested the effect of these expression constructs with different plant species, petunia and lettuce, which are receptive to *Agrobacterium*-mediated gene transfer. As a result, autonomous differentiation of calli and shoots from leaf explants cultured on the PGR-free medium was observed at 4WAI (**Supplementary Figure 6**). The presence of transgenes in the obtained hygromycin-resistant shoots was confirmed (**Supplementary Figure 7**).

The ectopic overexpression of DRs has been reported to cause morphogenic abnormalities in transgenic plants (Boutilier et al., 2002; Srinivasan et al., 2007; Chen et al., 2022b), and the higher expression level linked to severe phenotype in tobacco (Srinivasan et al., 2007). The obtained tobacco transgenic plants in the present study showed malformation with various intensities. The qRT-PCR of *BBM* and *WUS* in the plant lines showing ‘nearly normal’, ‘moderate,’ and ‘severe’ malformations showed similar co-relation between DRs levels and morphologies (**Supplementary Figure 8**).

### 3.2 Phytohormone contents in differentiating tobacco leaf cells

**Figure 2:**
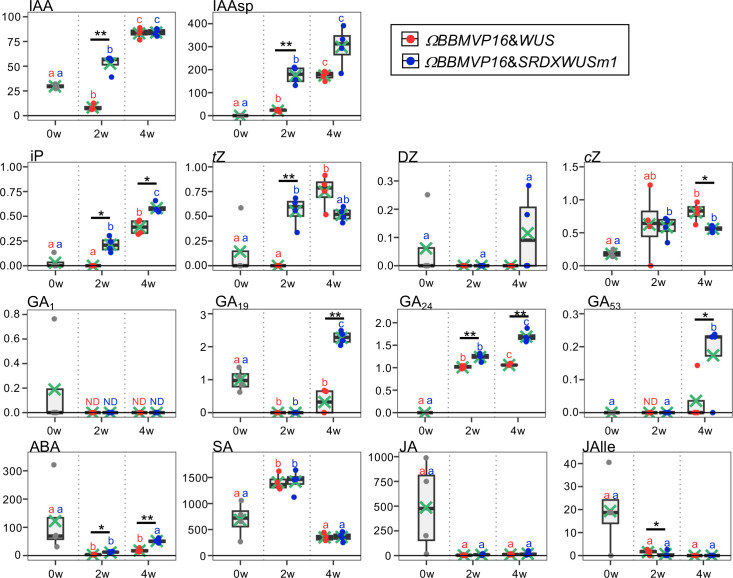
Phytohormone content in differentiating leaf explant cells with introduced *ΩBBMVP16*&*WUS* and *ΩBBMVP16*&*SRDXWUSm1*. The phytohormone content in the leaf explant cultures was measured at two and four weeks after *Agrobacterium* infection. The y-axis represents the concentration (pmol/g fresh weight (FW)) and the x-axis represents the culture period (0, 2, and 4 weeks (w)). Orange and blue dots represent the values of *ΩBBMVP16*&*WUS-* and *ΩBBMVP16*&*SRDXWUSm1*-introduced leaf explant cultures, respectively. Non-infected leaf explants were analyzed as 0-week cultured samples (0w), and the results are indicated by gray dots. Light green crosses and vertical black bars associated with boxplots represent the mean and standard deviation, respectively (n = 4). The horizontal black bar in each boxplot represents the median value. IAA, Indole-3-acetic acid; IAAsp, indole-3-acetyl-L-aspartic acid; iP, N6-(Δ2-isopentenyl) adenine; *t*Z, *trans*-Zeatin; DZ, dihydrozeatin; *c*Z, *cis*-Zeatin; GA, Gibberellin; ABA, Abscisic acid; SA, Salicylic acid; JA, Jasmonic acid; JAIle, Jasmonic acid with isoleucine. Asterisks indicate significant differences (*p < 0.05, **p < 0.01) between *ΩBBMVP16*&*WUS-* and *ΩBBMVP16*&*SRDXWUSm1*-introduced leaf explant cultures during the same culture period, as detected by Welch’s t-test. Different lowercase letters in the same color indicate significant differences (*p <* 0.05) among the three time points (0, 2, and 4w), as detected using the Tukey-Kramer’s test. ND, not detected (below the quantification limit). All measurement data are shown in **Supplementary Table 2**.

Cultured cells differentiating without external PGR treatment are suitable for analyses to obtain pure endogenous cellular behaviors. Phytohormones are important regulators of cell division and differentiation. Because the differentiation of leaf cells caused by the introduction of *ΩBBMVP16*&*WUS* and *ΩBBMVP16*&*SRDXWUSm1* showed dramatically different patterns (**Figure 1A**; **Supplementary Figures 1G, J**), the underlying hormonal behaviors were analyzed. Six phytohormones (auxins, cytokinins, GAs, ABA, SA, and JA) and their metabolites in the leaf explant cultures at 2 and 4WAI were measured (**Figure 2**; **Supplementary Table 2**).

The active auxin indole-3-acetic acid (IAA) and the irreversible catabolite indole-3-acetyl-L-aspartic acid (IAAsp) showed similar behaviors in the *ΩBBMVP16*&*WUS-* and *ΩBBMVP16*&*SRDXWUSm1*-introduced leaf cultures by 4WAI. At 2WAI, compared with non-infected (0-week cultured) leaf explants, the IAA content in *ΩBBMVP16*&*SRDXWUSm1* was significantly higher, whereas it was significantly lower in *ΩBBMVP16*&*WUS*. The IAA content was further elevated to a similar level by 4WAI in both *ΩBBMVP16*&*WUS*- and *ΩBBMVP16*&*SRDXWUSm1*-introduced leaf cultures. The IAAsp content in *ΩBBMVP16*&*SRDXWUSm1*-introduced leaf cultures was also higher than that in *ΩBBMVP16*&*WUS*-introduced leaf cultures, especially at 2WAI.

In comparison with the non-infected leaf explants, similar cytokinin content, except for dihydrozeatin (DZ), was observed in both *ΩBBMVP16*&*WUS* and *ΩBBMVP16*&*SRDXWUSm1*-introduced leaf cultures. At 2WAI, N6-(Δ2-isopentenyl) adenine (iP) content was yet negligible in *ΩBBMVP16*&*WUS*, whereas it was significantly higher in *ΩBBMVP16*&*SRDXWUSm1*. The iP elevations in both *ΩBBMVP16*&*WUS*- and *ΩBBMVP6*&*SRDXWUSm1*-introduced leaf cultures continued for 4WAI, and the overall contents in the *ΩBBMVP16*&*SRDXWUSm1*-introduced leaf cultures were significantly higher than those in *ΩBBMVP16*&*WUS*. An earlier elevation of *trans*-zeatin (*t*Z) was observed in *ΩBBMVP16*&*SRDXWUSm1* than in *ΩBBMVP16*&*WUS*, and the content level was maintained by 4WAI. Although *t*Z was negligible in the *ΩBBMVP16*&*WUS*-introduced leaf cultures at 2WAI, it reached a level similar to that of *ΩBBMVP16*&*SRDXWUSm1* at 4WAI. Elevated *cis*-zeatin (*c*Z) level was detected in *ΩBBMVP16*&*SRDXWUSm1* compared to the non-infected leaf cultures at 2WAI, and each level was maintained until 4WAI. *c*Z content at 4WAI was significantly higher in *ΩBBMVP16*&*WUS* than in *ΩBBMVP16*&*SRDXWUSm1*.

The content behaviors were various depending on the GA type. One of the active GAs, GA_1_, was maintained at negligible levels by 4WAI in both *ΩBBMVP16*&*WUS-* and *ΩBBMVP16*&*SRDXWUSm1*-introduced leaf cultures. Significantly higher levels of GA_19_, GA_24_, and GA_53_ were detected in the *ΩBBMVP16*&*SRDXWUSm1*-introduced leaf cultures at 4WAI than in those of *ΩBBMVP16*&*WUS*, whereas continuous elevation of GA_24_ was also observed in the *ΩBBMVP16*&*WUS*-introduced leaf cultures. The content of GA_4_, an active GA, was below the detection level in all samples analyzed (**Supplementary Table 2**).

Compared to the non-infected leaf explants, the ABA content was lower in the *ΩBBMVP16*&*WUS*-introduced leaf cultures throughout the four weeks of culture from infection. In contrast, the ABA level was elevated in the *ΩBBMVP16*&*SRDXWUSm1*-introduced leaf cultures at the 4WAI, showing overall higher levels than that of *ΩBBMVP16*&*WUS*.

No significant differences in SA, JA, and JA-Ile content were detected throughout the four weeks of culture in the comparison of *ΩBBMVP16*&*WUS-* and *ΩBBMVP16*&*SRDXWUSm1*-introduced leaf cultures. Statistically, the JA-Ile content at 2WAI was significantly lower in the *ΩBBMVP16*&*SRDXWUSm1*-introduced leaf cultures. Focusing on active auxin (IAA) and cytokinins (iP, *t*Z), each phytohormone level tended to increase earlier and was higher in the leaf explant cultures at 2WAI with *ΩBBMVP16*&*SRDXWUSm1*-introduced leaf cultures than in those with *ΩBBMVP16*&*WUS*.

### 3.3 Gene expression profiles in differentiating tobacco leaf cells

**Figure 3:**
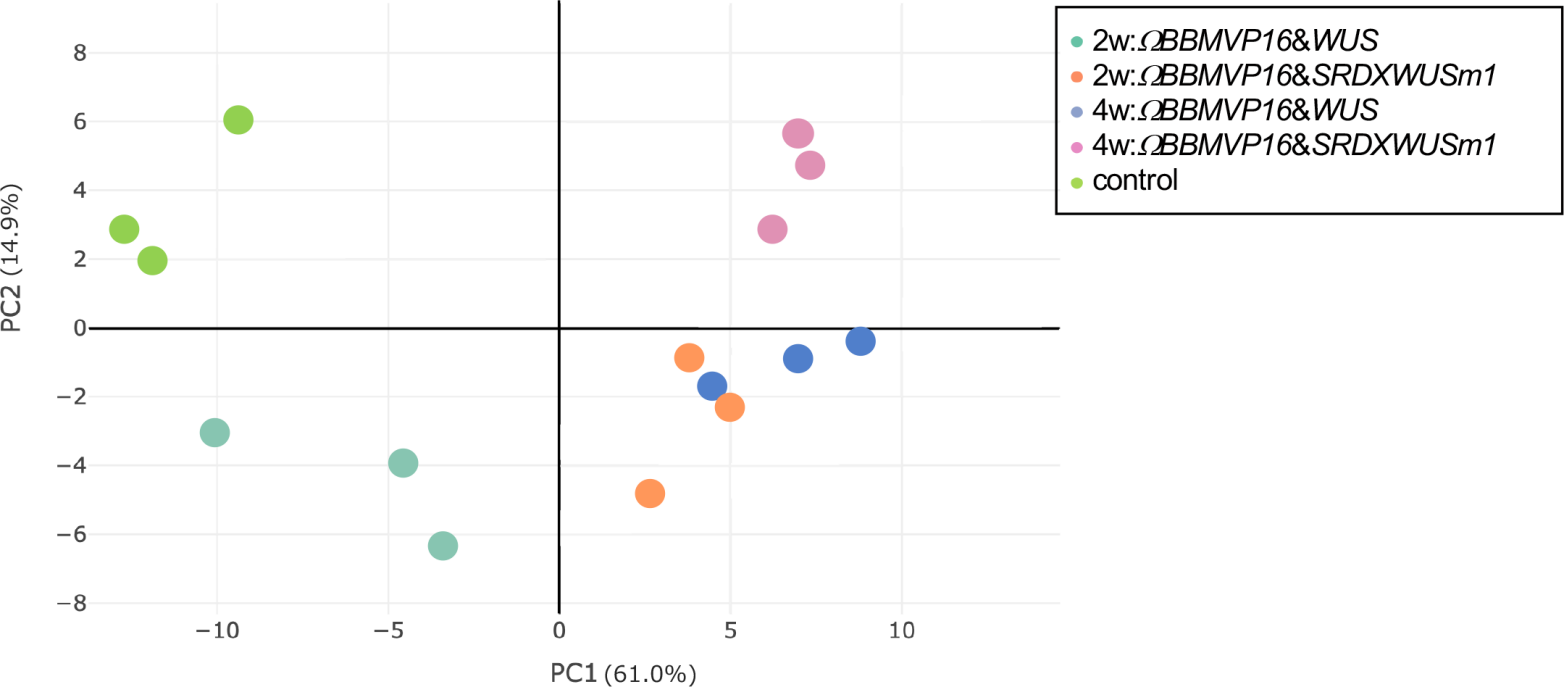
Principal component analysis of gene expression levels in tobacco leaf explant cultures. The ‘control’ is the experimental control (non-infected leaf explant). Plots with the same color indicate biological replicates. 2w and 4w, 2 (or 4) weeks after *Agrobacterium* infection.

To analyze transcriptional behavior during cell differentiation, we performed RNA-seq of tobacco leaf cells cultured for 2 or 4WAI with *ΩBBMVP16*&*WUS* and *ΩBBMVP16*&*SRDXWUSm1*, and 0-week cultured leaf explants without *Agrobacterium* infection (control). PCA showed that the three biological replicates of each sample represented by the same color clustered together (**Figure 3**). Notably, the groups of 2w:*ΩBBMVP16*&*SRDXWUSm1* and 4w:*ΩBBMVP16*&*WUS* were located close together.

**Figure 4:**
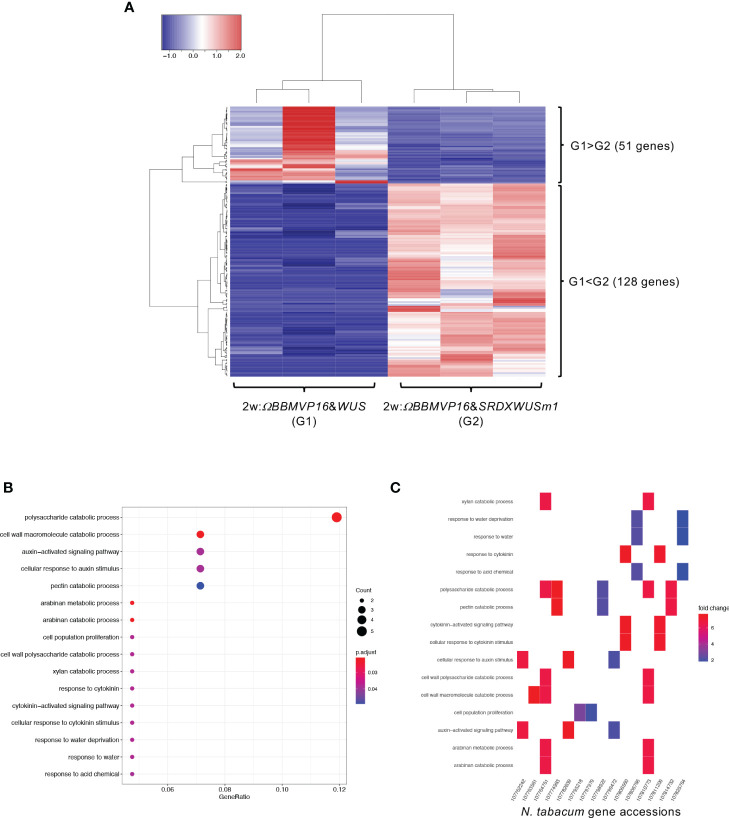
Differential expression analysis for two-group RNA-seq count data (2w:*ΩBBMVP16*&*WUS* vs 2w:*ΩBBMVP16*&*SRDXWUSm1*). Group1 (G1) is 2w:*ΩBBMVP16*&*WUS*, Group2 (G2) is 2w:*ΩBBMVP16*&*SRDXWUSm1.* **(A)** Hierarchical clustering heatmap of 179 DEGs (FDR < 0.05) generated using TCC-GUI. Red and blue indicate high and low gene expressions, respectively. **(B)** Visualization of GO enrichment analysis using 128 DEGs (G1 < G2). All detected 16 GO terms are shown. **(C)** *N. tabacum* gene accessions included 16 GO terms **(B)**. The color of the square represents the Log2 fold-change (M value of the MA plot).

First, we compared the gene expression of 2w:*ΩBBMVP16*&*WUS* (G1) and 2w:*ΩBBMVP16*&*SRDXWUSm1* (G2) (**Figure 4A**), where differences in the swiftness of differentiation and phytohormone levels were observed (**Figures 1A**, **2**; **Supplementary Figures 1G, J**). A total of 179 genes were identified as DEGs (FDR < 0.05). Among the DEGs, 128 genes were significantly upregulated in 2w:*ΩBBMVP16*&*SRDXWUSm1* (G1 < G2), whereas the expression of 51 genes was significantly upregulated in 2w:*ΩBBMVP16*&*WUS* (G1 > G2) (**Figure 4A**). GO analysis of the 128 DEGs (G1 < G2) detected 16 GO terms related to cell wall, auxin, cytokinin, and water (**Figure 4B**; **Supplementary Figure 9**). The putative *A. thaliana* orthologs for the detected genes are listed in **Supplementary Table 3**. Focusing on phytohormones, the genes categorized into auxin-related GO terms included orthologs of *AUXIN-RESPONSIBLE PROTEIN* (*AUX*/*IAAs*) (two DEGs) and *PIN-FORMED* (*PINs*) (one DEG) (**Figure 4C**; **Supplementary Table 3**). Genes categorized into the cytokinin-related GO were the orthologs of *HISTIDINE PHOSPHOTRANSFER PROTEIN 6* (*HP6*) (two DEGs) (**Figure 4C**; **Supplementary Table 3**). No significant GO terms were detected when using the 51 genes that were upregulated in 2w:*ΩBBMVP16*&*WUS*.

**Figure 5:**
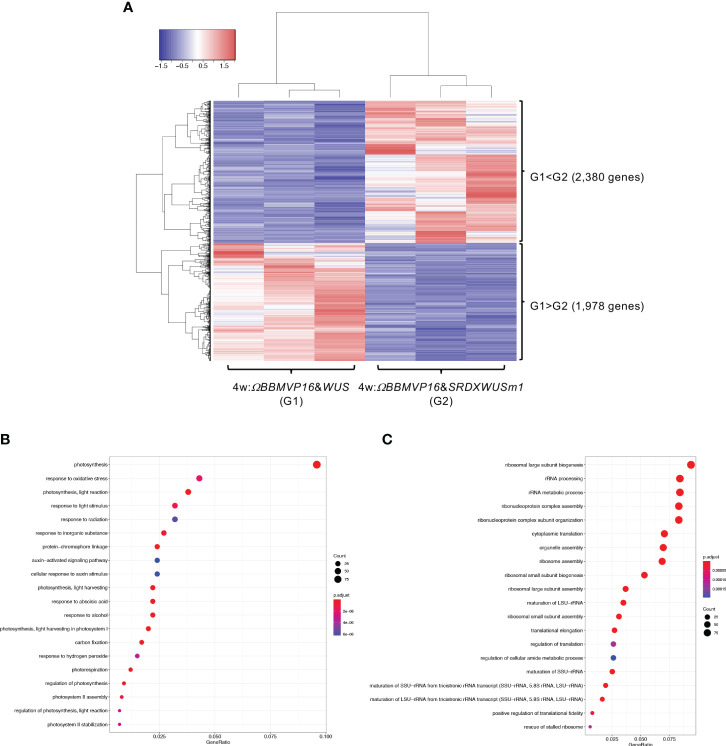
Differential expression analysis for two-group RNA-seq count data (4w:*ΩBBMVP16*&*WUS* vs 4w:*ΩBBMVP16*&*SRDXWUSm1*). Group1 (G1) is 4w:*ΩBBMVP16*&*WUS*, Group2 (G2) is 4w:*ΩBBMVP16*&*SRDXWUSm1.* **(A)** Hierarchical clustering heatmap of 4,358 DEGs (FDR < 0.05) generated using TCC-GUI. Red and blue indicate high and low gene expressions, respectively. **(B)** Visualization of GO enrichment analysis using 2,380 DEGs (G1 < G2). The top 20 GO terms are listed. **(C)** Visualization of GO enrichment analysis using 1,978 DEGs (G1 > G2). The top 20 GO terms are listed. All GO terms are shown in **Supplementary Figure 6**. The top 20 GO terms were selected based on FDR values for each comparison (G1 < G2 and G1 > G2).

We then compared gene expression between 4w:*ΩBBMVP16*&*WUS* (G1) and 4w:*ΩBBMVP16*&*SRDXWUSm1* (G2) (**Figure 5A**), where differences in the type of differentiation were observed (**Figure 1A**; **Supplementary Figures 1G, J**). Among the 4,358 genes detected as significant DEGs (FDR < 0.05), 2,380 genes were expressed at significantly higher levels in 4w:*ΩBBMVP16*&*SRDXWUSm1* (G1 < G2), whereas 1,978 genes were expressed at significantly lower levels (**Figure 5A**). With the 2,380 DEGs upregulated in 4w:*ΩBBMVP16*&*SRDXWUSm1*, GO terms related to photosynthesis were the most enriched, and GO terms related to auxin and ABA were also enriched (**Figure 5B**; **Supplementary Figures 10A**, **11**). In contrast, GO terms related to ribosomes were highly enriched in the downregulated 1,978 genes (**Figure 5C**; **Supplementary Figure 10B**). Focusing on the phytohormones, GO terms ‘auxin-activated signaling pathway’ and ‘cellular response to auxin stimulus’ included the orthologs of *AUX/IAAs* (10 DEGs), *AUXIN RESPONSES FACTORS* (*ARFs*) (6 DEGs), *PIN-likes* (*PILSs*) (3 DEGs), and *PIN6* (1 DEG) (**Supplementary Table 4**; **Supplementary Figure 12**). The GO term ‘response to ABA’ (**Figure 5B**) included orthologs of *LATE EMBRYOGENESIS ABUNDANT* (*LEAs*/*EMs*) (seven DEGs), *PYRABACTIN RESISTANCE-LIKE* (*PYLs*/*PYRs*/*RCARs*) (four DEGs), and *ABSCISIC ACID-INSENSITIVE 5* (*ABI5*) (two DEGs) (**Supplementary Table 4**; **Supplementary Figure 12**).

In summary, GO terms involved in phytohormones were enriched with genes upregulated by introducing *ΩBBMVP16*&*SRDXWUSm1* at 2 and 4WAI, compared to *ΩBBMVP16*&*WUS*. The GO terms related to phytohormones differed at each time point; GO terms related to auxin and cytokinin were enriched at 2WAI, whereas those related to auxin and ABA were enriched at 4WAI. These GO terms were associated with signaling or transport but not with biosynthesis and metabolism.

**Figure 6:**
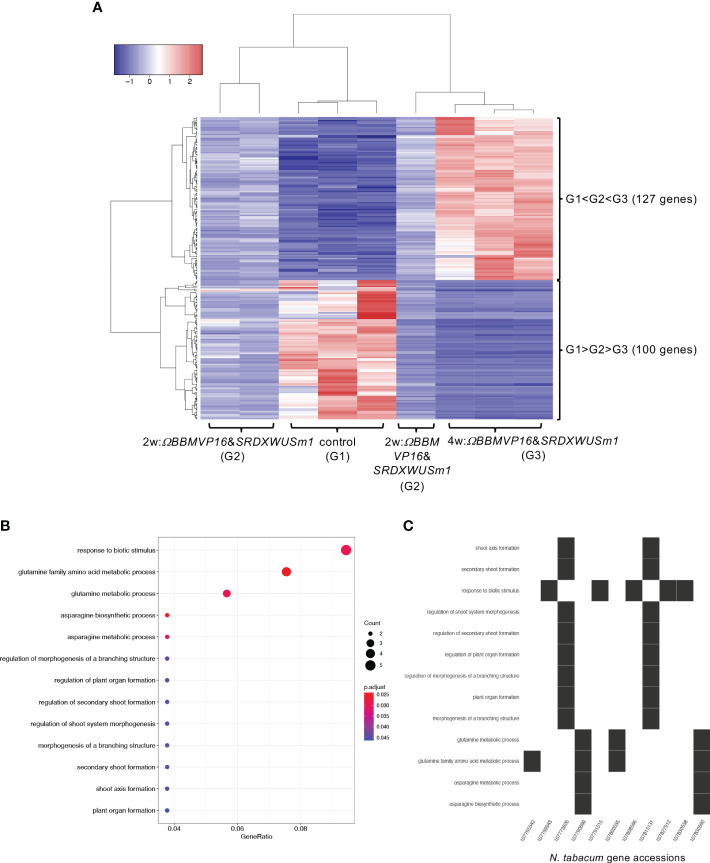
Differential expression analysis for three-group RNA-seq count data (control vs 2w:*ΩBBMVP16*&*SRDXWUSm1* vs 4w:*ΩBBMVP16*&*SRDXWUSm1*). Group1 (G1) is control (non-infected leaf explants), Group2 (G2) is 2w:*ΩBBMVP16*&*SRDXWUSm1*, and Group3 (G3) is 4w:*ΩBBMVP16*&*SRDXWUSm1.* **(A)** Hierarchical clustering heatmap of 117 genes identified as DEGs (FDR < 0.05) based on baySeq with TCC. Red and blue indicate high and low gene expressions, respectively. **(B)** Visualization of the GO enrichment analysis using 100 DEGs (G1 > G2 > G3). **(C)** *N. tabacum* gene accessions included 13 GO terms (G1 > G2 > G3).

Next, the RNA-seq data obtained from *ΩBBMVP16*&*SRDXWUSm1*-introduced leaf cells, which showed remarkable differentiation patterns for up to 4 weeks of culture, were analyzed by comparing the three groups: non-infected leaf explants (control: G1), 2w:*ΩBBMVP16*&*SRDXWUSm1* (G2), and 4w:*ΩBBMVP16*&*SRDXWUSm1* (G3) (**Figure 6A**). Among the 15,100 significant DEGs (FDR < 0.05), 117 genes were gradually upregulated (G1 < G2 < G3), whereas 100 genes were gradually downregulated (G1 > G2 > G3) during the culture process after *Agrobacterium* infection (**Figure 6A**). Although no significant GO terms (FDR < 0.05) were detected for the 117 upregulated genes, GO analysis of the 100 downregulated genes revealed 13 significant GO terms (FDR < 0.05) (**Figure 6B**; **Supplementary Figure 13**). Among the 100 downregulated genes, GO terms related to stimuli (five DEGs) included the ortholog genes of *OSMOTIN-LIKE34* (*OSM34*) and *MILDEW RESISTANCE LOCUS O* (*MLOs*). GO terms related to glutamine and asparagine (four DEGs) included *GLUTAMINE-DEPENDENT ASPARAGINE SYNTHASE 1* (*ASN1*), *GLUTAMINE SYNTHETASE 2* (*GS2*), and *GLUTAMATE DEHYDROGENASE* (*GDH*) (**Figure 6C**; **Supplementary Table 5**). In addition, eight GO terms (FDR < 0.05) related to shoot, morphogenesis, and organ formation were detected with two DEGs (LOC107773826 and LOC107815131) (**Figure 6C**), including the orthologs of *TEOSINTE BRANCHED1*/*CYCLOIDEA*/*PCF3*/*4*/*10* (*TCP3*/*4*/*10*) (**Supplementary Table 5**).

In a three-group comparison using *ΩBBMVP16*&*WUS*-introduced leaf cultures at 2WAI (G2) and 4WAI (G3) and non-infected leaf explants (control: G1) (**Supplementary Figure 14A**), 9,161 genes in total were identified as DEGs (FDR < 0.05). Among the 9,161 significant DEGs (FDR < 0.05), only two genes were upregulated (G1 < G2 < G3), whereas 74 genes were downregulated (G1 > G2 > G3) during the culture process after *Agrobacterium* infection (**Supplementary Figure 14A**). The 28 GO terms related to stimuli, metabolism, and catabolism were significantly (FDR < 0.05) enriched in the 74 downregulated genes (**Supplementary Figure 14B**). Because GO enrichment analysis could not be applied to only the two upregulated genes, we obtained each gene description from NCBI; LOC107761077 was annotated as the ortholog of *AT-HOOK MOTIF NUCLEAR-LOCALIZED PROTEIN 15* (*AHL15*/*AGF2*), and LOC107809734 was an uncharacterized gene (**Supplementary Table 6**).

The DEGs detected in this study were summarized as Venn diagram (**Supplementary Figure 15**); significantly upregulated in *ΩBBMVP16*&*SRDXWUSm1*-introduced leaf cells at both 2 (**Figure 4**) and 4WAI (**Figure 5**), and gradually upregulated during the culture process in *ΩBBMVP16*&*SRDXWUSm1*-introduced leaf cells (**Figure 6**). Among 19 genes commonly detected as significantly upregulated in *ΩBBMVP16*&*SRDXWUSm1*-introduced leaf cells at both 2 (blue) and 4WAI (green in **Supplementary Figure 15**), cell proliferation and auxin-related GO terms were included (FDR < 0.05) (**Supplementary Table 7**). In this analysis, one common gene LOC107801243 was detected in the three-group comparison (purple in **Supplementary Figure 15**; **Supplementary Table 7**). On the other hand, no significant GO terms were detected for the intersect DEGs that were downregulated (data not shown).

### 3.4 qRT-PCR of the genes detected as DEGs in *ΩBBMVP16*&*SRDXWUSm1*-introduced leaf cell cultures

**Figure 7:**
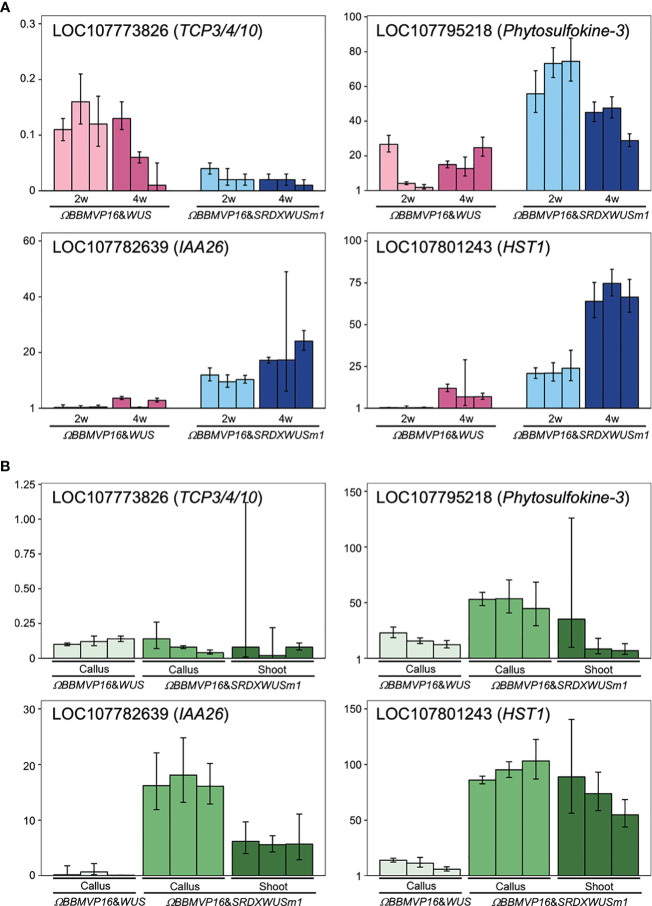
Relative expression levels of four genes detected as DEGs by *ΩBBMVP16*&*SRDXWUSm1* introduction. **(A)** q-RT PCR analysis with samples derived from three biological replicates of infection with *ΩBBMVP16*&*WUS* and *ΩBBMVP16*&*SRDXWUSm1*. The x-axis represents the 2 or 4-week-cultured leaf explant cultures (2w and 4w) introduced with each construct. **(B)** qRT-PCR with transgenic calli and shoots samples. The shoots introduced with *ΩBBMVP16*&*WUS* were not analyzed because of the scarce formation (**Figure 1B**). Three independent samples detached from the differentiating leaf explant cell culture were analyzed. The y-axis in all graphs represents the relative quantification (RQ; 2^-ΔΔCt^) compared to the control (non-infected leaf explant). The error bars represent the maximum and minimum RQ values of three reaction replicates (95.0% confidence level).

To evaluate the actual expression behaviors of DEGs affected by *ΩBBMVP16*&*SRDXWUSm1* introduction, qRT-PCR was performed for four genes: LOC107773826 down-regulated over time (**Supplementary Table 5**), LOC107782639 and LOC107795218 showed higher levels compared to *ΩBBMVP16*&*WUS* (**Supplementary Table 7**), and LOC107801243 upregulated over time and higher compared to *ΩBBMVP16*&*WUS* (**Supplementary Table 7**). LOC107773826 and LOC107782639 are the putative orthologs of Arabidopsis *TCP3/4/10* and *IAA26*, respectively (**Supplementary Tables 3**-**5**). The LOC107795218 is annotated as a putative phytosulfokine-3 gene (XM_016617810). The LOC107801243 is identified as a putative paralogue gene of NAD^+^-dependent protein deacetylase HST1 (XP_016480848), as detected by tBLASTn search (NCBI). Expression analysis revealed that LOC107773826 (*TCP3/4/10*) was consistently downregulated in all infected leaf cell cultures at 2 and 4WAI compared to the control (non-infected leaf explant). Notably, expression levels were lower in leaf cells introduced with the *ΩBBMVP16*&*SRDXWUSm1* than in those with *ΩBBMVP16*&*WUS* (**Figure 7A**). Both LOC107782639 (*IAA26*) and LOC107795218 (*Phytosulfokine-3*) showed higher expression levels in *ΩBBMVP16*&*SRDXWUSm1*-introduced leaf cells than in *ΩBBMVP16*&*WUS*-introduced cells at 2 and 4 WAI (**Figure 7A**). LOC107801243 (*HST1*) showed high expression in the *ΩBBMVP16*&*SRDXWUSm1*-introduced leaf cell cultures, further upregulated at 4WAI (**Figure 7A**). These qRT-PCR results corresponded with DEGs identified from RNA-seq analysis.

Furthermore, qRT-PCR of the same four genes was performed for transgenic calli and shoots derived from leaf explant cultures. Compared to the non-infected leaf explants (control), LOC107773826 (*TCP3/4/10*) expression was downregulated in all tested calli and shoots (**Figure 7B**). In general, expression levels of LOC107782639 (*IAA26*), LOC107795218 (*Phytosulfokine-3*), and LOC107801243 (*HST1*) were higher in*ΩBBMVP16*&*SRDXWUSm1* samples compared to *ΩBBMVP16*&*WUS*, particularly in callus (**Figure 7B**).

## 4 Discussion

### 4.1 Modification of *Arabidopsis BBM* and *WUS* enabled autonomous cell differentiation of the transgenic cells without external PGR treatment

In the present study, we demonstrated that the combined expression of *Arabidopsis BBM* and *WUS* in transgenic tobacco, petunia, and lettuce leaf cells resulted in autonomous cell differentiation without the application of PGRs during culture process (**Figure 1A**; **Supplementary Figures 1**-**7**). Single and combined introduction of *BBM* and *WUS*, which are regulated by the 35S and RPS5A promoters, respectively, was insufficient to induce cell differentiation (**Supplementary Figures 1B, D, F**). A single introduction of *ΩBBMVP16*, in which the translation enhancer omega sequence and the VP16 transcriptional activator were applied, was still insufficient (**Supplementary Figure 1C**). In addition, a single introduction of *SRDXWUSm1*, in where two amino acid mutations were inserted into the WUS-box and an artificial strong repression domain, SRDX, was fused to the N-terminal end of WUSm1, did not induce cell differentiation (**Supplementary Figure 1E**). In contrast, the combined expression of *ΩBBMVP16* and *WUS* (*ΩBBMVP16*&*WUS*) successfully induced transgenic calli (**Figure 1A**; **Supplementary Figure 1G**). In a previous study, *Arabidopsis* and *Brassica napus BBM* were evaluated in tobacco, and inducible differentiation from leaf explant cells was achieved by the supportive application of cytokinins, and leaves from the *35S::BBM* transgenic tobacco plants did not cause cellular differentiation in PGR-free culture conditions (Srinivasan et al., 2007). Taken together with the previous insights and our results, the single expression of translationally and functionally enhanced *BBM* could alter the cell physiology involved in endogenous phytohormone for differentiation; however, it might still be insufficient to cause morphological alterations as apparent differentiation. As for *WUS*, the expression of *Arabidopsis WUS* induces adventitious shoot formation from the root tissues in *Arabidopsis* and tobacco (Rashid et al., 2007; Ikeda et al., 2009, 2020). Therefore, to induce adventitious shoot differentiation from somatic cells by the ectopic expression of WUS, it may be critical that somatic cells have a root-pertaining genetic background. Thus, the differentiation achieved by the combined expression of *ΩBBMVP16* and *WUS* (or *SRDXWUS, SRDXWUSm1*) suggests that these DRs synergistically influence gene expression and physiology, resulting in cellular differentiation. In leaf cell cultures exhibiting autonomous differentiation, relatively lower levels of *WUS* expression were observed compared to *BBM* expression levels (**Supplementary Figure 3**). This observation supports the speculation that WUS triggers cell proliferation and organogenesis with substantial physiological modifications by BBM. The malformation patterns observed in the regenerated transgenic plants were associated with higher DR expression levels, particularly a significant *BBM* level (**Supplementary Figure 8**). To regulate the morphology of regenerated plants, employing inducible promoters would improve the current system.

The combined expression of *ΩBBMVP16* and *SRDXWUSm1* (or *SRDXWUS*) showed a different differentiation pattern compared to *ΩBBMVP16*&*WUS*, showing faster and more accelerated shoot differentiation in tobacco transgenic cells (**Figure 1A**; **Supplementary Figures 1I, J**). A similar tendency was observed in the case of petunia and lettuce (**Supplementary Figure 6**). The ectopic expression of *SRDXWUSm1* is more effective than *WUS* in inducing adventitious shoot formation from somatic cells in *Arabidopsis* (Ikeda et al., 2020). Similarly, our study demonstrated a significant effect on autonomous shoot organogenesis in tobacco cells through the expression of *SRDXWUS* or *SRDXWUSm1* in combination with *ΩBBMVP16*, indicating that SRDX fusion to the N-terminal of WUS was effective for the cellular differentiation also in tobacco cell (**Supplementary Figures 1**, **2**). Regarding the WUS-box mutation, WUSm1 showed a reduced ability for autonomous callus formation compared to WUS (**Supplementary Figures 1G, H**, **2**). In contrast, SRDXWUSm1 induced higher callus formation than SRDXWUS (**Supplementary Figure 2**). WUS function as a repressor in maintaining pluripotent stem cells, and WUS-box is crucial for this role (Ikeda et al., 2009). It has been suggested that the fusion of the artificial strong repression domain SRDX to the WUSm1 generates a protein with enhanced repression activity, thereby promoting organogenesis through cytokinin signaling (Ikeda et al., 2020). Alternatively, it could be postulated that the acceleration of stem cell maintenance repression disruption by the WUS-box mutation was facilitated by SRDX fusion, thereby stimulating cell division in tobacco cells.

### 4.2 Phytohormone behaviors underlining dedifferentiation and redifferentiation

Because the present *BBM* and *WUS* expression system enabled autonomous cell differentiation on a PGR-free medium, pure endogenous phytohormone behaviors underlying cellular differentiation could be analyzed. To understand the swiftness and differences in cell differentiation observed in this study, phytohormone-quantitative analysis was performed (**Figure 2**; **Supplementary Table 2**). Active auxin (IAA) and cytokinin (iP and *t*Z) levels were higher in 2w:*ΩBBMVP16*&*SRDXWUSm1* and 4w:*ΩBBMVP16*&*WUS*, in which callus induction was observed. The timing of the increase in active auxin/cytokinin content coincided with callus induction in tobacco leaf explants (**Figure 1A**). This result is consistent with the acknowledged theory that increased auxin and cytokinin levels in tissue culture medium promote dedifferentiation (Skoog and Miller, 1957; Phillips and Garda, 2019).

Focusing on 4WAI, the calli that emerged from *ΩBBMVP16*&*SRDXWUSm1* induced a more shoot-like structure, indicating that redifferentiation was promoted compared to *ΩBBMVP16*&*WUS*. The contents of GA_19_, GA_24_, and GA_53_, which are precursors of active GA_1_ or GA_4_ (He et al., 2020), were significantly higher in *ΩBBMVP16*&*SRDXWUSm1* than in *ΩBBMVP16*&*WUS*, whereas all the active GAs (GA_1_, GA_3,_ GA_4_, and GA_7_) were not present at the quantitative level in any of the analyzed samples (**Figure 2**; **Supplementary Table 2**). Active GAs negatively regulate shoot regeneration in *Arabidopsis* (Ezura and Harberd, 1995). Active GAs and cytokinins act antagonistically during shoot regeneration in tobacco (Engelke et al., 1973). In our study, the accumulation of active GA precursors (**Figure 2**) suggests that active GAs synthesis was suppressed and endogenous cytokinins were not antagonized for shoot regeneration in *ΩBBMVP16*&*SRDXWUSm1*-introduced leaf cells.

In the present study, a significant increase in ABA was observed in 4w:*ΩBBMVP16*&*SRDXWUSm1*. Although the substantial contribution of ABA to cellular differentiation has not yet been elucidated (Shin et al., 2020), ABA is known to crosstalk with other phytohormones, and is involved in plant growth regulation and stress (Skubacz et al., 2016). Thus, the observed elevation suggests the involvement of ABA in organogenesis in the coordinated balance between auxin and cytokinin that maintained this level.

### 4.3 Gene expression differences between the differentiated cells caused by *ΩBBMVP16*&*WUS* and *ΩBBMVP16*&*SRDXWUSm1*

In RNA-seq analysis, GO terms such as ‘auxin-activated signaling pathways’ and ‘response to cytokinin’ were enriched with the DEGs upregulated in 2w:*ΩBBMVP16*&*SRDXWUSm1* (**Figures 4B, C**; **Supplementary Figure 9**). These GO terms included the putative orthologs of *AUX/IAAs* and *PIN1/3/4/7* (**Supplementary Table 3**), which coincided with increased levels of active auxin (**Figure 2**). The other detected ortholog in the GO terms, *Arabidopsis* HP6, has been known to act as an inhibitor of cytokinin signaling (Mähönen et al., 2006; Müller and Sheen, 2007; Besnard et al., 2014). Moreover, it has also been reported that overexpression of *HP6* in SAM suppresses type-A ARRs, which repress cytokinin signaling (Besnard et al., 2014). In our study, the tobacco orthologous gene of *Arabidopsis HP6* was upregulated in 2w:*ΩBBMVP16*&*SRDXWUSm1* more than in 2w:*ΩBBMVP16*&*WUS*. The calli induced by *ΩBBMVP16*&*SRDXWUSm1* had greenish organ-like structures (**Figure 1A**; **Supplementary Figure 1J**). Therefore, it was suggested that upregulation of *HP6* promoted the suppression of type-A ARRs, resulting in the activation of cytokinin signaling, cell division, and near-organogenic differentiation. Active cytokinin content was also higher in 2w:*ΩBBMVP16*&*SRDXWUSm1* than in 2w:*ΩBBMVP16*&*WUS* (**Figure 2**), which is consistent with this hypothesis. Previous studies have reported that *HP6* is directly activated by auxin (Bishopp et al., 2011; Besnard et al., 2014) and affects the localization of PIN1 in *Arabidopsis* (Moreira et al., 2013). Such interactions of HP6 with auxin and PINs may be involved in the differences in the differentiation phenotypes caused by *ΩBBMVP16*&*WUS* and *ΩBBMVP16*&*SRDXWUSm1*. In addition, GO terms related to the cell wall, especially the ‘polysaccharide catabolic process’ were significantly upregulated in 2w:*ΩBBMVP16*&*SRDXWUSm1* compared to 2w:*ΩBBMVP16*&*WUS* (**Figures 4B, C**; **Supplementary Table 3**). A previous study reported that WUS homeobox-containing 13 (WOX13) is a key regulator to promote callus formation by modifying the cell wall properties in *Arabidopsis* (Ikeuchi et al., 2022). WOX13 directly upregulates cell wall-related genes involved in the polysaccharide catabolic process (Ikeuchi et al., 2022). WOX13 conserves the WUS-homeodomain and an acidic domain but lacks the WUS-box (Dolzblasz et al., 2016) and targets sequences that also target *WUS* (Ma et al., 2019; Ikeuchi et al., 2022). The promoted callus formation by SRDXWUSm1 observed in our study (**Supplementary Figure 2**) could reflect a similar activation to WOX13.

The 4w:*ΩBBMVP16*&*SRDXWUSm1* leaf cells differentiated into calli and shoots, in contrast to 4w:*ΩBBMVP16*&*WUS*, in which only callus formation was observed (**Figure 1A**; **Supplementary Figures 1G, J**). The result that the active cytokinin iP content was higher (**Figure 2**) was reminiscent of the differentiation tendency of *N. tabacum* in tissue culture, where a higher ratio of cytokinin to auxin induces organogenesis (Skoog and Miller, 1957; Phillips and Garda, 2019). Although the GO terms related to cytokinin were not enriched, GO terms related to auxin and ABA were detected in the DEG upregulated in 4w:*ΩBBMVP16*&*SRDXWUSm1* (**Figure 5B**; **Supplementary Figures 10A**, **11**; **Supplementary Table 4**). In addition to auxin transport, response, and signaling, GO terms related to auxins included accessions involved in shoot development (**Figure 5B**; **Supplementary Figures 10A**, **11**; **Supplementary Table 4**). This result may reflect the adventitious shoot formation observed in the present study (**Figure 1A**; **Supplementary Figure 1J**). Although the role of ABA in plant regeneration has not been extensively studied, several studies have suggested its effects on shoot regeneration (Shin et al., 2020). For example, the leucine-rich repeat receptor-like kinase *RECEPTOR-LIKE PROTEIN KINASE1* (*RPK1*), which is involved in ABA signaling, promotes shoot regeneration in *Arabidopsis* (Motte et al., 2014). As mentioned above, ABA is also known to crosstalk with other phytohormones (Skubacz et al., 2016), as reported that ABA-induced ABI5 inhibited PINs accumulation and auxin activity, for instance (Yuan et al., 2014). The fact that many GO terms related to stress were detected (**Figure 5B**; **Supplementary Figure 10A**) implies the involvement of ABA. In our study, the putative orthologs of *ABI5* and *PINs* were detected as DEG that were upregulated in 4w:*ΩBBMVP16*&*SRDXWUSm1* (**Supplementary Table 4**), suggesting that phytohormone crosstalk, especially by auxin and ABA, was influenced by organogenesis, such as shoot formation.

The primary GO terms detected in DEGs downregulated in 4w:*ΩBBMVP16*&*SRDXWUSm1* were related to metabolism, which is likely involved in transcription and translation (**Figure 5C**; **Supplementary Figure 10B**). This result reflects the dominance of the above metabolic pathways for active cell division in callus formation (4w:*ΩBBMVP16*&*WUS*) rather than for organogenesis (4w:*ΩBBMVP16*&*SRDXWUSm1*).

### 4.4 Changes in gene expression over time in *ΩBBMVP16*&*SRDXWUSm1*-introduced leaf cells

No significant changes in the expression of genes related to phytohormones or regeneration were observed over time in *ΩBBMVP16*&*WUS*-introduced leaf cells, whereas GO terms related to the regulation of plant organogenesis were significantly downregulated over time in *ΩBBMVP16*&*SRDXWUSm1*-introduced leaf cells. Genes related to the regulation of plant organogenesis were identified as the *TCP3/4/10* orthologs (**Supplementary Table 5**), and qRT-PCR also showed the significant downregulation in *ΩBBMVP16*&*SRDXWUSm1*-introduced leaf cells and differentiated calli and shoots (**Figure 7**). TCP4 is involved in multiple plant developmental processes, such as leaf and flower morphogenesis, secondary cell wall biosynthesis, senescence, and hormone signaling in *Arabidopsis* (Palatnik et al., 2003; Schommer et al., 2008; Nag et al., 2009; Sarvepalli and Nath, 2011; Li et al., 2012; Sun et al., 2017). A previous study reported promotion of shoot regeneration in *tcp3/4/10* mutant in *Arabidopsis*, indicating that TCPs are strong negative regulators of *de novo* shoot regeneration (Yang et al., 2020). The downregulation of *TCP3/4/10* over time in *ΩBBMVP16*&*SRDXWUSm1*-introduced leaf cell cultures might account for the enhanced differentiation, while the significant difference in the expression level between differentiated calli and shoots was not observed in our study (**Figure 7B**). In addition, GO terms related to glutamine and asparagine were gradually downregulated in *ΩBBMVP16*&*SRDXWUSm1*-introduced leaf cells (**Figure 6B**; **Supplementary Figure 13**), and the ortholog genes of *GDHs*, *GS2*, and *ASN1* were annotated (**Supplementary Table 5**). These genes are upregulated in the dark to activate nitrogen assimilation (Yoneyama and Suzuki, 2020; Liu et al., 2022). In the present study, chloroplast differentiation was observed in *ΩBBMVP16*&*SRDXWUSm1*-introduced leaf cells (**Figure 1A**; **Supplementary Figure 1J**), and all culture processes were performed under continuous fluorescent light. These results and the environmental conditions may account for the downregulation of these genes. In addition, GO terms related to stimuli were detected from five DEGs, including *OSM34* and *MLOs* orthologs, which have been reported to be involved in the ABA response (Lim and Lee, 2014; Park and Kim, 2021). The relationship between the elevated ABA level at 4WAI compared to that at 2WAI (**Figure 2**) and the downregulation of these ortholog genes cannot be explained at present, although it could imply that altered ABA signaling influenced cellular differentiation.

The present study identified 19 genes as the common intersections among DEGs upregulated during culture over time by *ΩBBMVP16*&*SRDXWUSm1* compared to *ΩBBMVP16*&*WUS*. Among these genes, LOC107801243 was upregulated compared to both the control and *ΩBBMVP16*&*WUS* (**Supplementary Figure 15**; **Supplementary Table 7**). The qRT-PCR analysis of selected genes LOC107782639 (*IAA26*), LOC107795218 (*Phytosulfokine-3*), and LOC107801243 (NAD^+^-dependent protein deacetylase HST1) showed similar behavior to those detected by RNA-seq, showing upregulation in both differentiated calli and shoots (**Figure 7**). The IAA26 belongs to the Aux/IAA transcription factor family and is known to respond to auxin. Therefore, the increased levels of auxins (**Figure 2**) may have contributed to the upregulation of LOC107782639. Phytosulfokine is a plant peptide growth factor that affects plant growth and differentiation (Ding et al., 2022). A study has shown that altered expression of an Arabidopsis phytosulfokine receptor influenced the callus formation capacity in response to PGRs (Matsubayashi et al., 2006). Considering the higher expression of LOC107795218 (*Phytosulfokine-3*) was observed in calli induced with *ΩBBMVP16*&*SRDXWUSm1* (**Figure 7B**), enhanced LOC107795218 expression may be associated with callus induction. In the yeast *Saccharomyces cerevisiae*, it has been reported that NAD^+^-dependent protein deacetylase HST1 is a sensor of cellular NAD^+^ levels involved in homeostasis (Bedalov et al., 2003). NAD^+^ is converted from nicotinic acid imported from the culture medium. Additionally, yeast HST1 represses the expression of thiamin biosynthesis genes by binding to their promoters, thereby regulating thiamin homeostasis (Li et al., 2010). Nicotinic acid and thiamin are components of the MS medium. These findings and our results (**Figure 7**; **Supplementary Figure 15**; **Supplementary Table 7**) suggest that LOC107801243 was highly upregulated in actively differentiating cells importing extracellular vitamins. In our study, no orthologs of *LEC1*, *LEC2*, *ABSCISIC ACIDINSENSITIVE3* (*ABI3*), and *FUSCA3* (*FUS3*), which are known to be activated by *BBM* (Horstman et al., 2017), were detected. The present study examined DEGs resulting from the combined expression of *SRDXWUS* or *SRDXWUSm1* with *ΩBBMVP16*. It is possible that these combinations could have influenced gene networks differently compared to the single expression of *BBM* alone. Future functional analysis of found DEGs and investigation of gene networks related to known DRs would provide further insights into cellular differentiation and organogenesis.

## 5 Conclusion

In this study, we demonstrated the functional efficacy of co-expressing Brassicaceae Arabidopsis BBM and WUS genes in inducing autonomous cellular differentiation with some plant cultivars from the Solanaceae and Asteraceae families. The functional enhancement by fusing VP16 to BBM and the functional modification of the WUS by the addition of SRDX showed drastic effect on accelerating the differentiation of transgenic cells toward organogenesis. The ectopic expressions of these DRs consequently influenced phytohormone levels and gene expressions involved in auxin response, metabolism, and organogenesis, resulting in fine-tuned physiology for differentiation. A noteworthy aspect of this study is the complete omission of external PGR application during tissue and cell culture for plant regeneration, a typically prerequisite process for transformation and genome-editing studies. Further investigations on the functional conservation of BBM and WUS in different plant species would improve the versatility of this system and enable its simplification. Challenges to develop an accurate regulation system for gene expression and the levels are also ongoing.

## Data availability statement

The datasets presented in this study can be found in online repositories. The names of the repository/repositories and accession number(s) can be found below: Bioproject accession number: PRJNA1049661, https://dataview.ncbi.nlm.nih.gov/object/PRJNA1049661?reviewer=r5siqhguq12lgug1tqlrkmfmk3.

## Author contributions

YS: Investigation, Methodology, Writing – original draft. MM: Data curation, Writing – review & editing. SK: Investigation, Writing – original draft. BP: Investigation, Writing – original draft. MK: Investigation, Writing – review & editing. YT: Investigation, Writing – review & editing. HS: Investigation, Writing – review & editing. TI: Conceptualization, Funding acquisition, Methodology, Supervision, Writing – review & editing, Writing – original draft.
