# Supplementary material for: Autonomous differentiation of transgenic cells requiring no external hormone application: the endogenous gene expression and phytohormone behaviors
Source: Front Plant Sci. 2024 Apr 3;15:1308417. doi: 10.3389/fpls.2024.1308417 (PMC11021773; doi:10.3389/fpls.2024.1308417)
Supplement: Supplementary file 1 [file DataSheet_1.zip › Data Sheet 1 (1)/Figures S1 - S15.PPTX]

## Slide 1
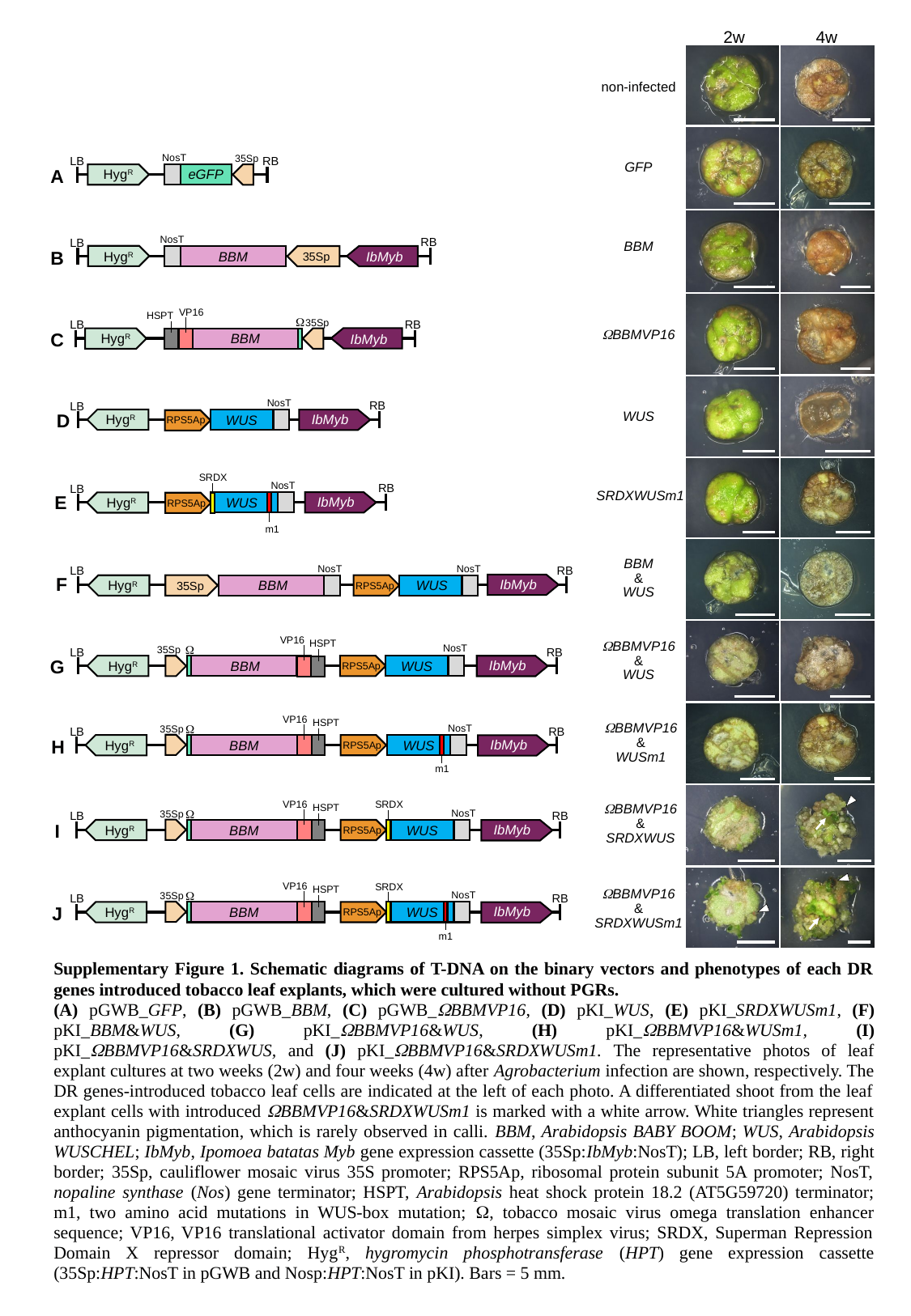

2w
4w
non-infected
NosT
35Sp
RB
HygR
eGFP
LB
A
GFP
NosT
RB
IbMyb
HygR
BBM
35Sp
LB
B
BBM
 VP16
HSPT
Ω
35Sp
RB
IbMyb
BBM
HygR
LB
C
ΩBBMVP16
NosT
RB
IbMyb
HygR
RPS5Ap
WUS
LB
D
WUS
SRDX
NosT
RB
IbMyb
WUS
HygR
RPS5Ap
LB
E
m1
 SRDXWUSm1
BBM
&
WUS
NosT
NosT
RB
IbMyb
BBM
WUS
HygR
35Sp
RPS5Ap
LB
F
 VP16
HSPT
NosT
Ω
35Sp
RB
IbMyb
BBM
WUS
HygR
RPS5Ap
LB
G
ΩBBMVP16
&
WUS
 VP16
HSPT
Ω
NosT
35Sp
LB
RB
H
IbMyb
BBM
WUS
HygR
RPS5Ap
m1
ΩBBMVP16
&
WUSm1
 VP16
SRDX
HSPT
Ω
NosT
35Sp
LB
RB
I
IbMyb
BBM
WUS
HygR
RPS5Ap
ΩBBMVP16
&
SRDXWUS
 VP16
SRDX
HSPT
Ω
NosT
35Sp
LB
RB
J
IbMyb
BBM
WUS
HygR
RPS5Ap
m1
ΩBBMVP16
&
SRDXWUSm1
Supplementary Figure 1. Schematic diagrams of T-DNA on the binary vectors and phenotypes of each DR genes introduced tobacco leaf explants, which were cultured without PGRs.
(A) pGWB_GFP, (B) pGWB_BBM, (C) pGWB_WBBMVP16, (D) pKI_WUS, (E) pKI_SRDXWUSm1, (F) pKI_BBM&WUS, (G) pKI_WBBMVP16&WUS, (H) pKI_WBBMVP16&WUSm1, (I) pKI_WBBMVP16&SRDXWUS, and (J) pKI_WBBMVP16&SRDXWUSm1. The representative photos of leaf explant cultures at two weeks (2w) and four weeks (4w) after Agrobacterium infection are shown, respectively. The DR genes-introduced tobacco leaf cells are indicated at the left of each photo. A differentiated shoot from the leaf explant cells with introduced WBBMVP16&SRDXWUSm1 is marked with a white arrow. White triangles represent anthocyanin pigmentation, which is rarely observed in calli. BBM, Arabidopsis BABY BOOM; WUS, Arabidopsis WUSCHEL; IbMyb, Ipomoea batatas Myb gene expression cassette (35Sp:IbMyb:NosT); LB, left border; RB, right border; 35Sp, cauliflower mosaic virus 35S promoter; RPS5Ap, ribosomal protein subunit 5A promoter; NosT, nopaline synthase (Nos) gene terminator; HSPT, Arabidopsis heat shock protein 18.2 (AT5G59720) terminator; m1, two amino acid mutations in WUS-box mutation; W, tobacco mosaic virus omega translation enhancer sequence; VP16, VP16 translational activator domain from herpes simplex virus; SRDX, Superman Repression Domain X repressor domain; HygR, hygromycin phosphotransferase (HPT) gene expression cassette (35Sp:HPT:NosT in pGWB and Nosp:HPT:NosT in pKI). Bars = 5 mm.

## Slide 2
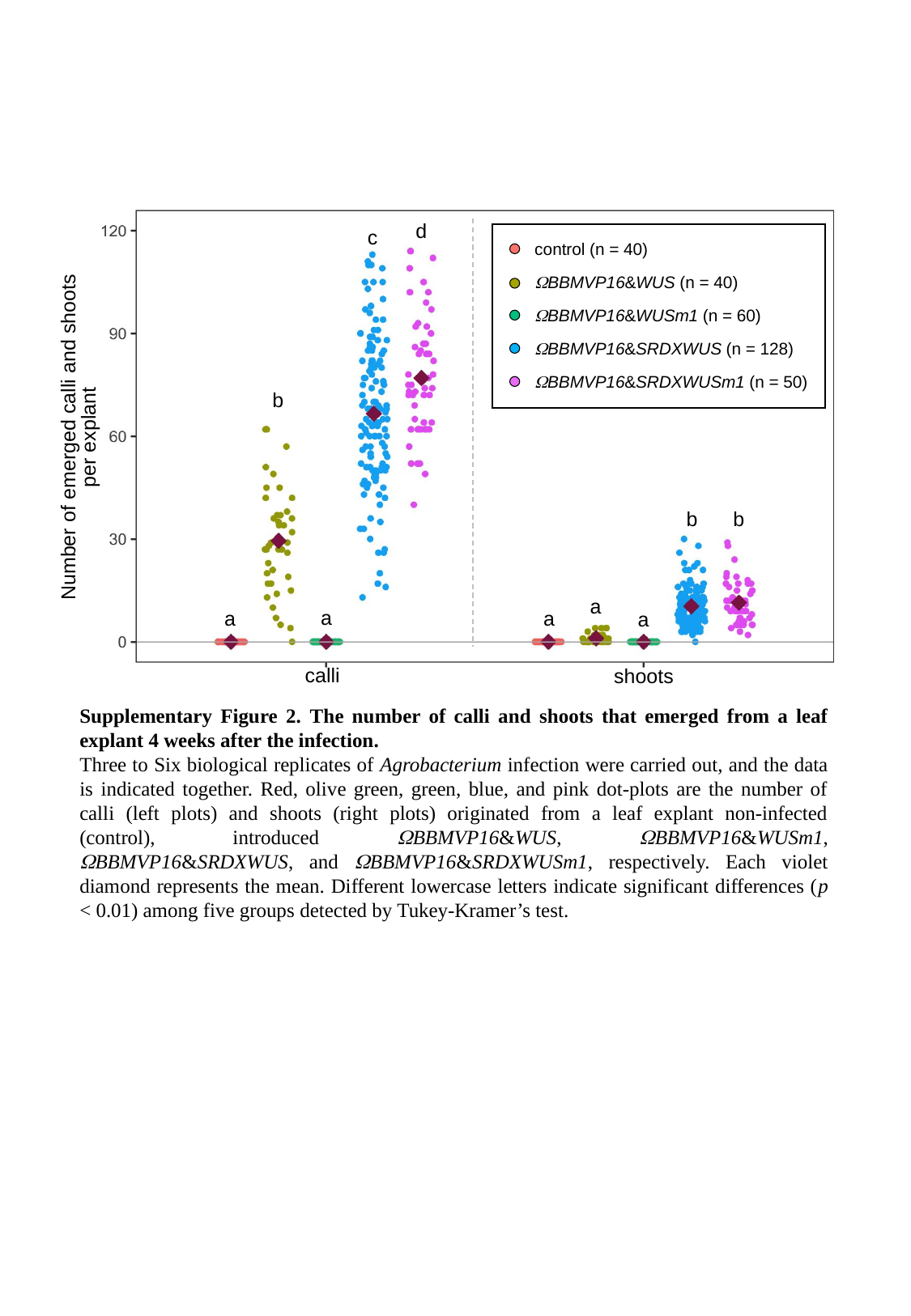

d
c
b
b
b
a
a
a
a
a
control (n = 40)
ΩBBMVP16&WUS (n = 40)
ΩBBMVP16&WUS (n = 40)
ΩBBMVP16&WUSm1 (n = 60)
ΩBBMVP16&SRDXWUS (n = 128)
ΩBBMVP16&SRDXWUSm1 (n = 50)
Number of emerged calli and shoots per explant
calli
shoots
Supplementary Figure 2. The number of calli and shoots that emerged from a leaf explant 4 weeks after the infection.
Three to Six biological replicates of Agrobacterium infection were carried out, and the data is indicated together. Red, olive green, green, blue, and pink dot-plots are the number of calli (left plots) and shoots (right plots) originated from a leaf explant non-infected (control), introduced ΩBBMVP16&WUS, ΩBBMVP16&WUSm1, ΩBBMVP16&SRDXWUS, and ΩBBMVP16&SRDXWUSm1, respectively. Each violet diamond represents the mean. Different lowercase letters indicate significant differences (p < 0.01) among five groups detected by Tukey-Kramer’s test.

## Slide 3
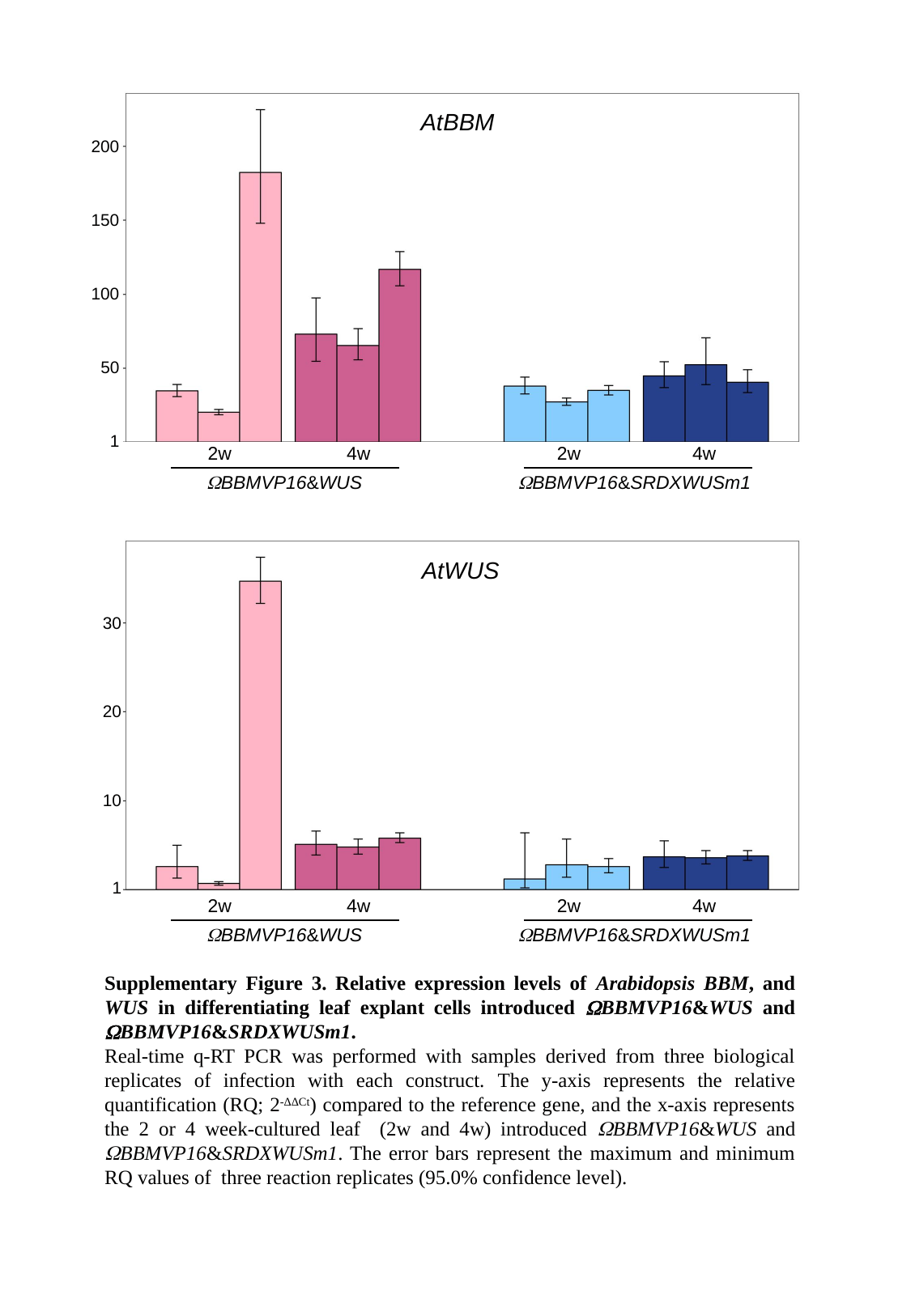

AtBBM
200
150
100
50
1
2w
4w
2w
4w
ΩBBMVP16&WUS
ΩBBMVP16&SRDXWUSm1
AtWUS
30
20
10
1
2w
4w
2w
4w
ΩBBMVP16&WUS
ΩBBMVP16&SRDXWUSm1
Supplementary Figure 3. Relative expression levels of Arabidopsis BBM, and WUS in differentiating leaf explant cells introduced ΩBBMVP16&WUS and ΩBBMVP16&SRDXWUSm1.
Real-time q-RT PCR was performed with samples derived from three biological replicates of infection with each construct. The y-axis represents the relative quantification (RQ; 2-ΔΔCt) compared to the reference gene, and the x-axis represents the 2 or 4 week-cultured leaf (2w and 4w) introduced ΩBBMVP16&WUS and ΩBBMVP16&SRDXWUSm1. The error bars represent the maximum and minimum RQ values of three reaction replicates (95.0% confidence level).

## Slide 4
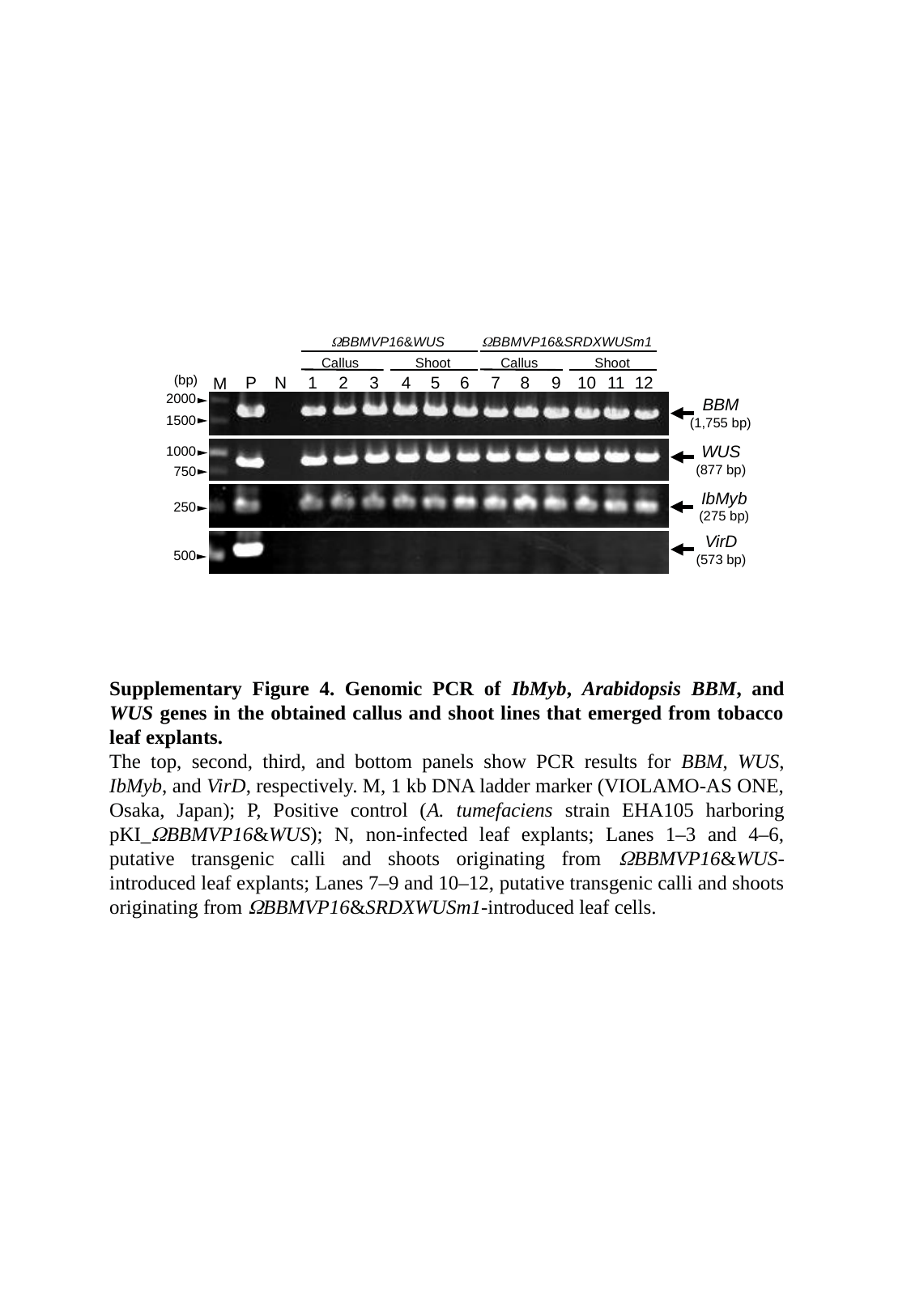

ΩBBMVP16&WUS
Shoot
Callus
ΩBBMVP16&SRDXWUSm1
Shoot
Callus
(bp)
12
P
N
1
2
3
4
5
6
7
8
9
10
11
M
2000
BBM
(1,755 bp)
1500
WUS
(877 bp)
1000
750
IbMyb
(275 bp)
250
VirD
(573 bp)
500
Supplementary Figure 4. Genomic PCR of IbMyb, Arabidopsis BBM, and WUS genes in the obtained callus and shoot lines that emerged from tobacco leaf explants.
The top, second, third, and bottom panels show PCR results for BBM, WUS, IbMyb, and VirD, respectively. M, 1 kb DNA ladder marker (VIOLAMO-AS ONE, Osaka, Japan); P, Positive control (A. tumefaciens strain EHA105 harboring pKI_WBBMVP16&WUS); N, non-infected leaf explants; Lanes 1–3 and 4–6, putative transgenic calli and shoots originating from WBBMVP16&WUS-introduced leaf explants; Lanes 7–9 and 10–12, putative transgenic calli and shoots originating from WBBMVP16&SRDXWUSm1-introduced leaf cells.

## Slide 5
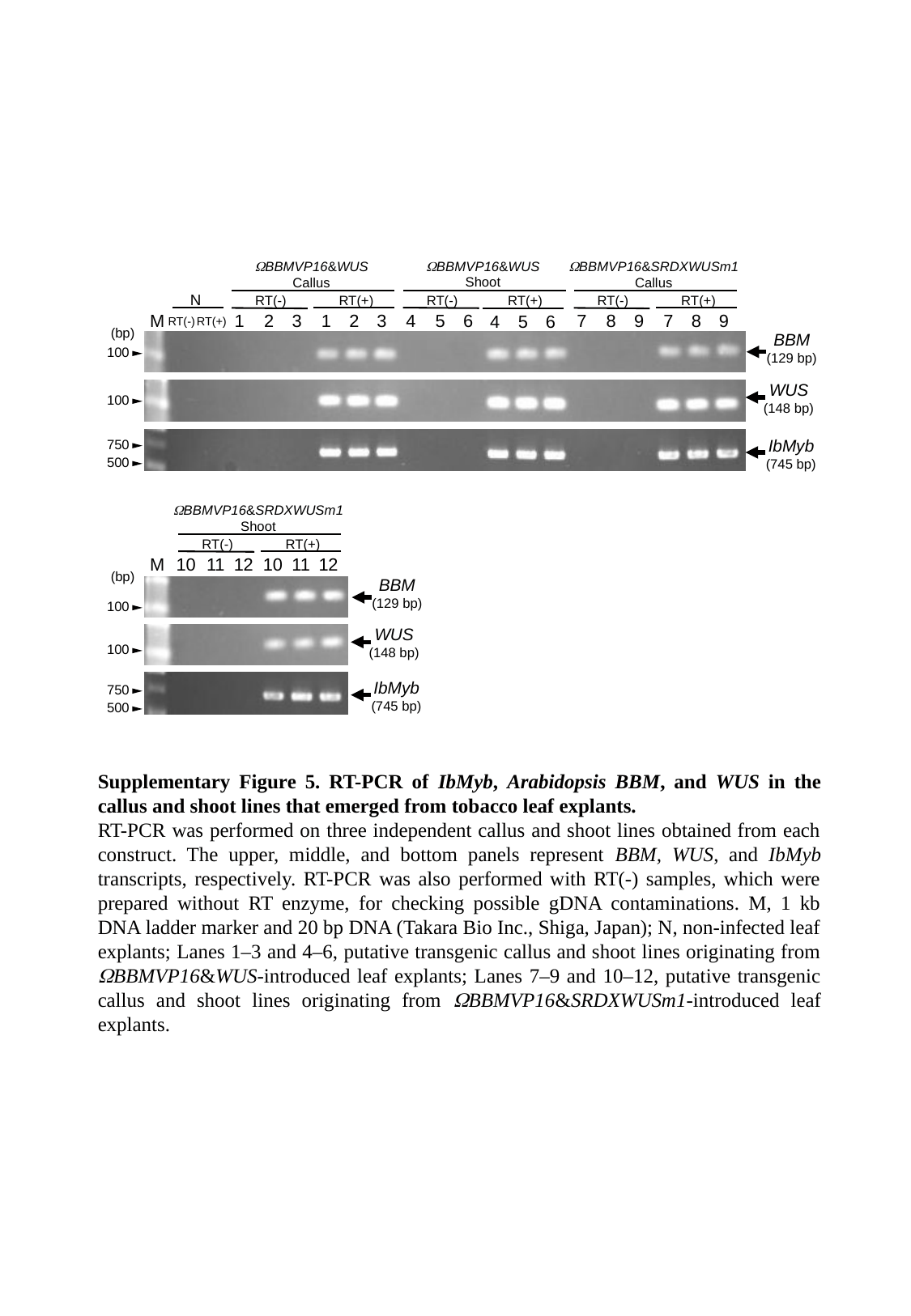

ΩBBMVP16&WUS
Shoot
RT(-)
RT(+)
4
5
6
4
5
6
ΩBBMVP16&WUS
Callus
RT(-)
RT(+)
1
2
3
1
2
3
ΩBBMVP16&SRDXWUSm1
Callus
RT(-)
RT(+)
7
8
9
7
8
9
N
M
RT(-)
RT(+)
(bp)
BBM
(129 bp)
100
WUS
(148 bp)
100
IbMyb
(745 bp)
750
500
ΩBBMVP16&SRDXWUSm1
Shoot
RT(-)
RT(+)
10
11
12
10
11
12
M
(bp)
BBM
(129 bp)
100
WUS
(148 bp)
100
IbMyb
(745 bp)
750
500
Supplementary Figure 5. RT-PCR of IbMyb, Arabidopsis BBM, and WUS in the callus and shoot lines that emerged from tobacco leaf explants.
RT-PCR was performed on three independent callus and shoot lines obtained from each construct. The upper, middle, and bottom panels represent BBM, WUS, and IbMyb transcripts, respectively. RT-PCR was also performed with RT(-) samples, which were prepared without RT enzyme, for checking possible gDNA contaminations. M, 1 kb DNA ladder marker and 20 bp DNA (Takara Bio Inc., Shiga, Japan); N, non-infected leaf explants; Lanes 1–3 and 4–6, putative transgenic callus and shoot lines originating from WBBMVP16&WUS-introduced leaf explants; Lanes 7–9 and 10–12, putative transgenic callus and shoot lines originating from WBBMVP16&SRDXWUSm1-introduced leaf explants.

## Slide 6
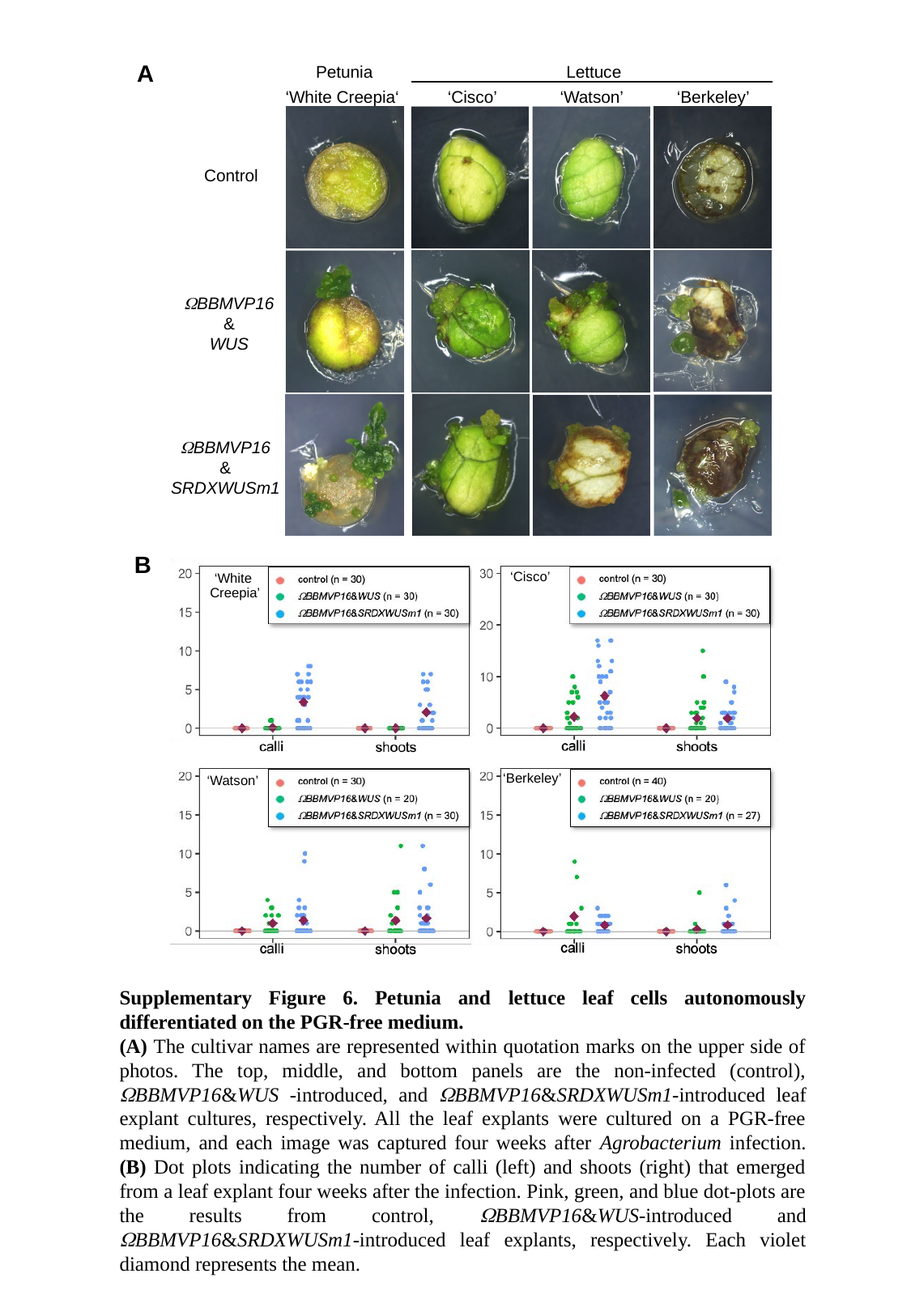

A
Petunia
Lettuce
‘White Creepia‘
‘Cisco’
‘Watson’
‘Berkeley’
Control
WBBMVP16
&
WUS
WBBMVP16
&
SRDXWUSm1
B
‘Cisco’
‘White
Creepia’
‘Berkeley’
‘Watson’
Supplementary Figure 6. Petunia and lettuce leaf cells autonomously differentiated on the PGR-free medium.
(A) The cultivar names are represented within quotation marks on the upper side of photos. The top, middle, and bottom panels are the non-infected (control), WBBMVP16&WUS -introduced, and WBBMVP16&SRDXWUSm1-introduced leaf explant cultures, respectively. All the leaf explants were cultured on a PGR-free medium, and each image was captured four weeks after Agrobacterium infection. (B) Dot plots indicating the number of calli (left) and shoots (right) that emerged from a leaf explant four weeks after the infection. Pink, green, and blue dot-plots are the results from control, WBBMVP16&WUS-introduced and WBBMVP16&SRDXWUSm1-introduced leaf explants, respectively. Each violet diamond represents the mean.

## Slide 7
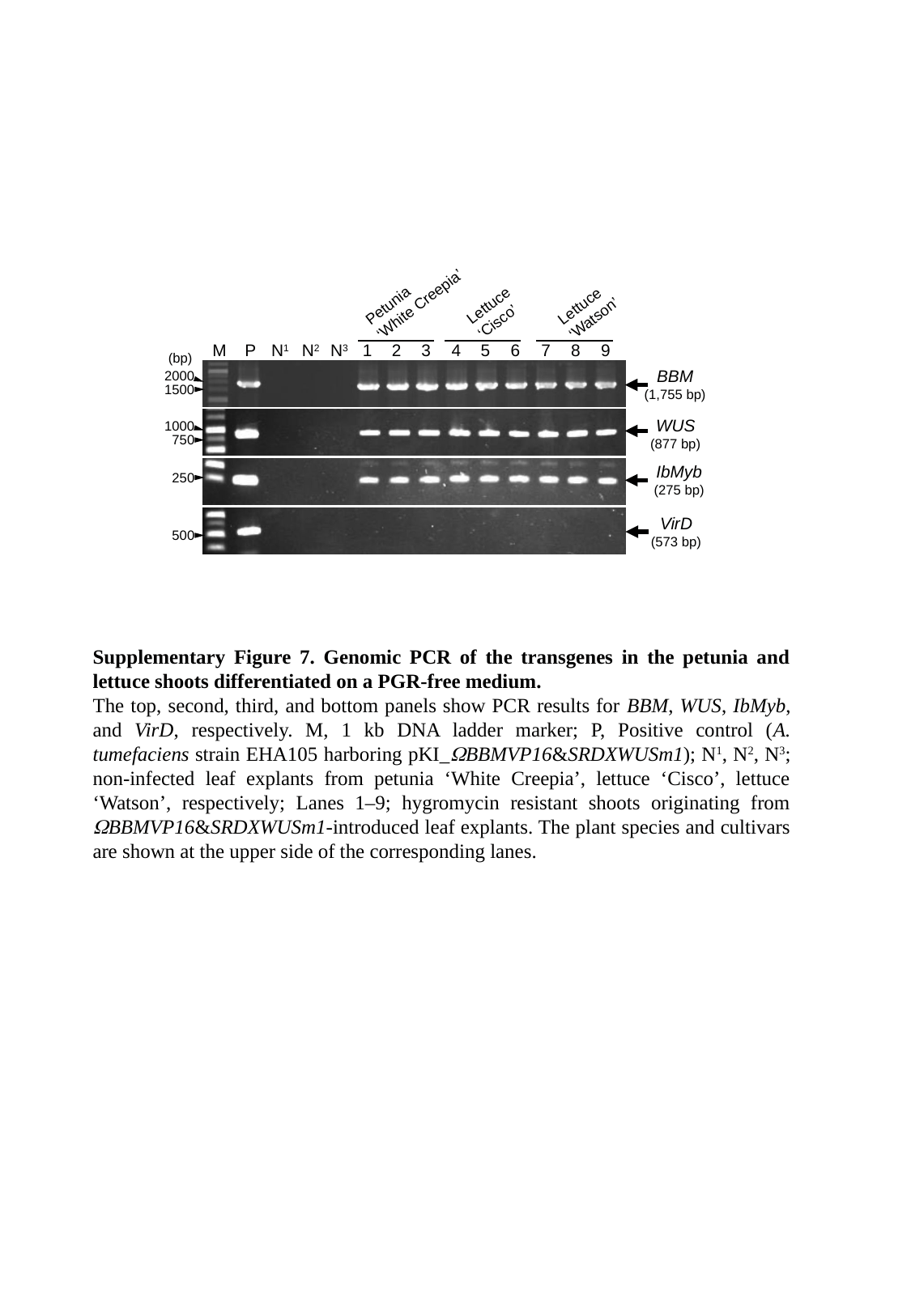

Petunia
‘White Creepia’
Lettuce
‘Watson’
Lettuce
‘Cisco’
1
2
3
4
5
6
7
8
9
P
N1
M
N3
N2
(bp)
2000
1500
1000
750
250
500
BBM
(1,755 bp)
WUS
(877 bp)
IbMyb
(275 bp)
VirD
(573 bp)
Supplementary Figure 7. Genomic PCR of the transgenes in the petunia and lettuce shoots differentiated on a PGR-free medium.
The top, second, third, and bottom panels show PCR results for BBM, WUS, IbMyb, and VirD, respectively. M, 1 kb DNA ladder marker; P, Positive control (A. tumefaciens strain EHA105 harboring pKI_WBBMVP16&SRDXWUSm1); N1, N2, N3; non-infected leaf explants from petunia ‘White Creepia’, lettuce ‘Cisco’, lettuce ‘Watson’, respectively; Lanes 1–9; hygromycin resistant shoots originating from WBBMVP16&SRDXWUSm1-introduced leaf explants. The plant species and cultivars are shown at the upper side of the corresponding lanes.

## Slide 8
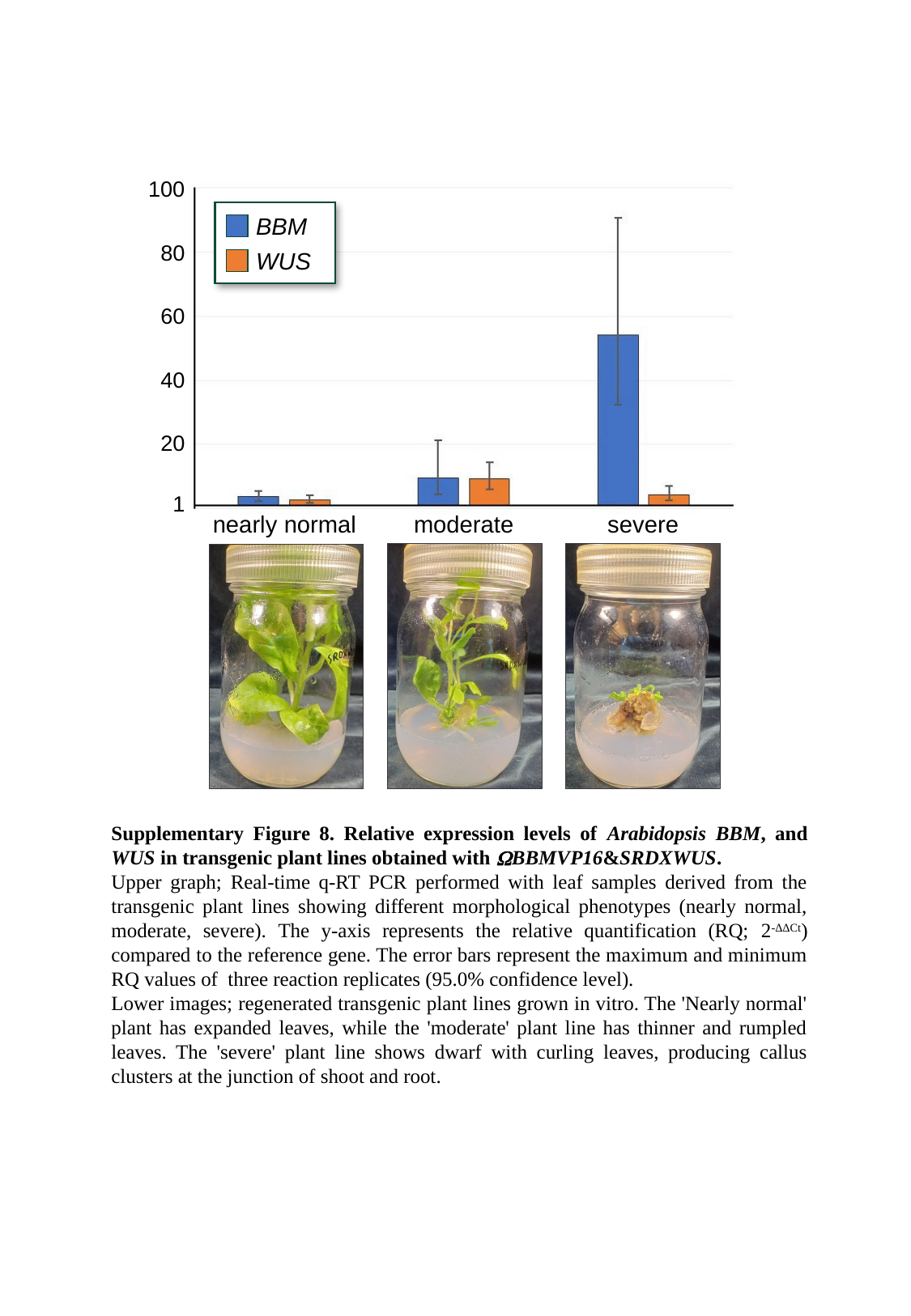

100
80
60
40
20
1
BBM
WUS
nearly normal
moderate
severe
Supplementary Figure 8. Relative expression levels of Arabidopsis BBM, and WUS in transgenic plant lines obtained with ΩBBMVP16&SRDXWUS.
Upper graph; Real-time q-RT PCR performed with leaf samples derived from the transgenic plant lines showing different morphological phenotypes (nearly normal, moderate, severe). The y-axis represents the relative quantification (RQ; 2-ΔΔCt) compared to the reference gene. The error bars represent the maximum and minimum RQ values of three reaction replicates (95.0% confidence level).
Lower images; regenerated transgenic plant lines grown in vitro. The 'Nearly normal' plant has expanded leaves, while the 'moderate' plant line has thinner and rumpled leaves. The 'severe' plant line shows dwarf with curling leaves, producing callus clusters at the junction of shoot and root.

## Slide 9
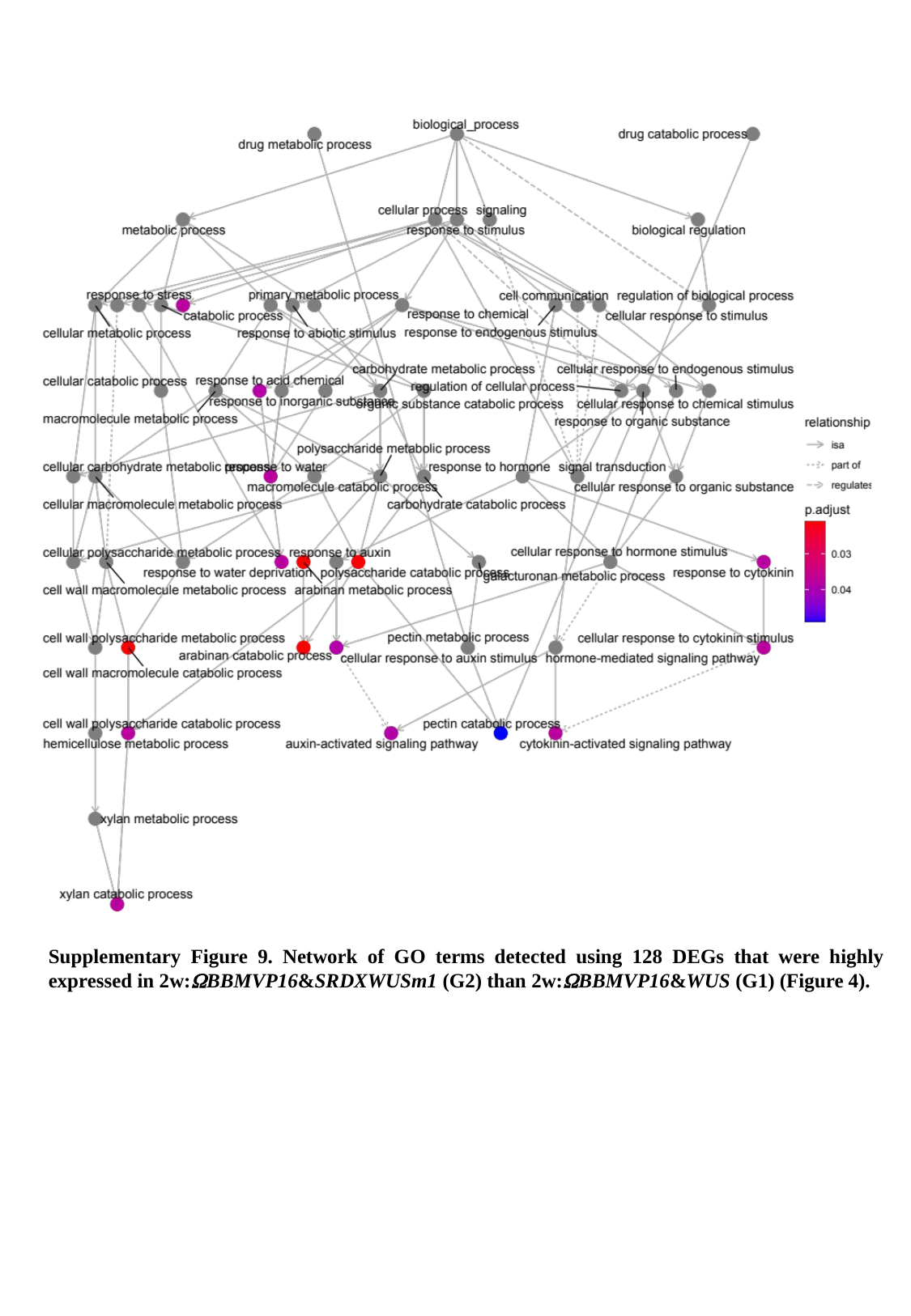

Supplementary Figure 9. Network of GO terms detected using 128 DEGs that were highly expressed in 2w:WBBMVP16&SRDXWUSm1 (G2) than 2w:WBBMVP16&WUS (G1) (Figure 4).

## Slide 10
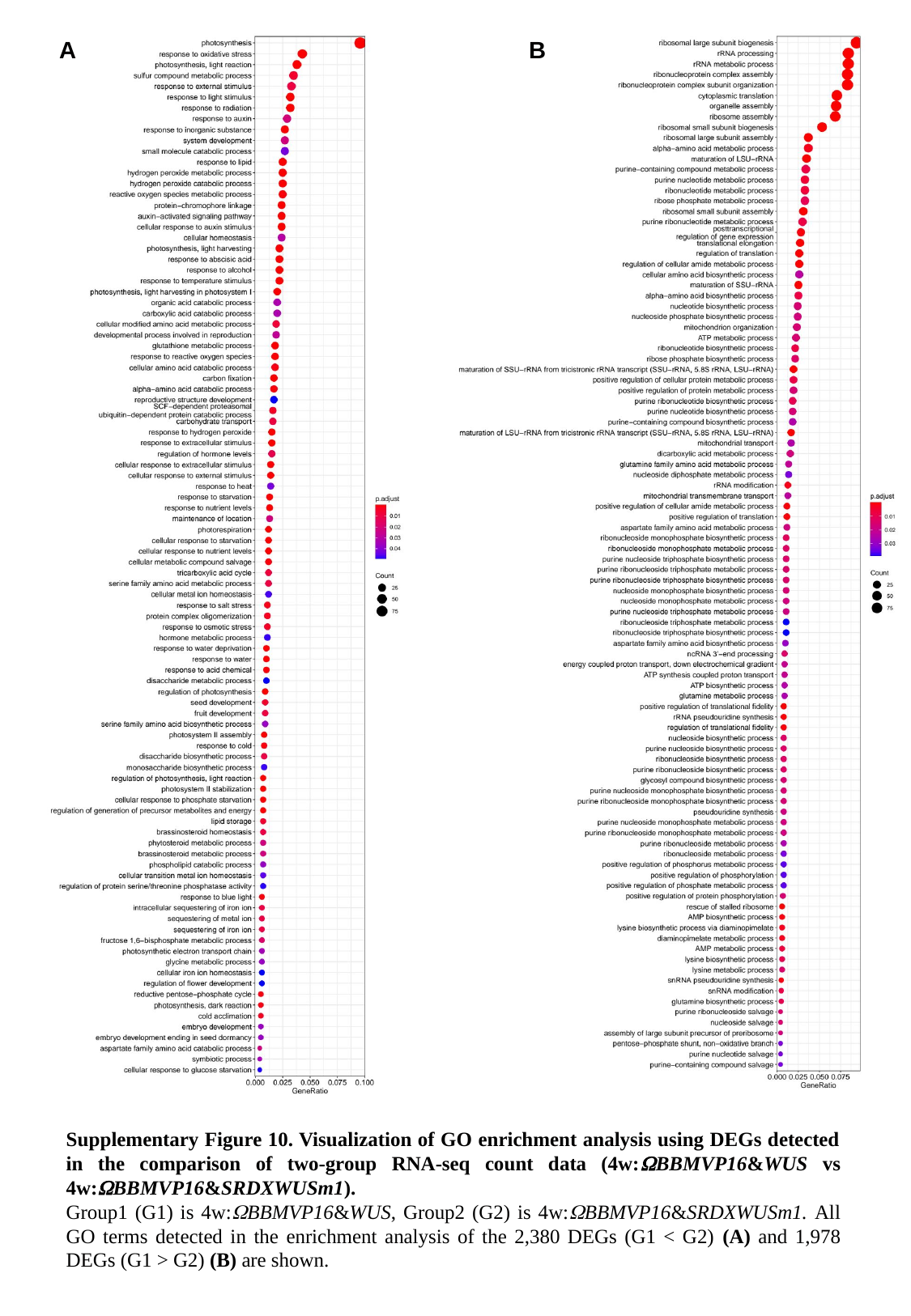

A
B
Supplementary Figure 10. Visualization of GO enrichment analysis using DEGs detected in the comparison of two-group RNA-seq count data (4w:WBBMVP16&WUS vs 4w:WBBMVP16&SRDXWUSm1).
Group1 (G1) is 4w:WBBMVP16&WUS, Group2 (G2) is 4w:WBBMVP16&SRDXWUSm1. All GO terms detected in the enrichment analysis of the 2,380 DEGs (G1 < G2) (A) and 1,978 DEGs (G1 > G2) (B) are shown.

## Slide 11
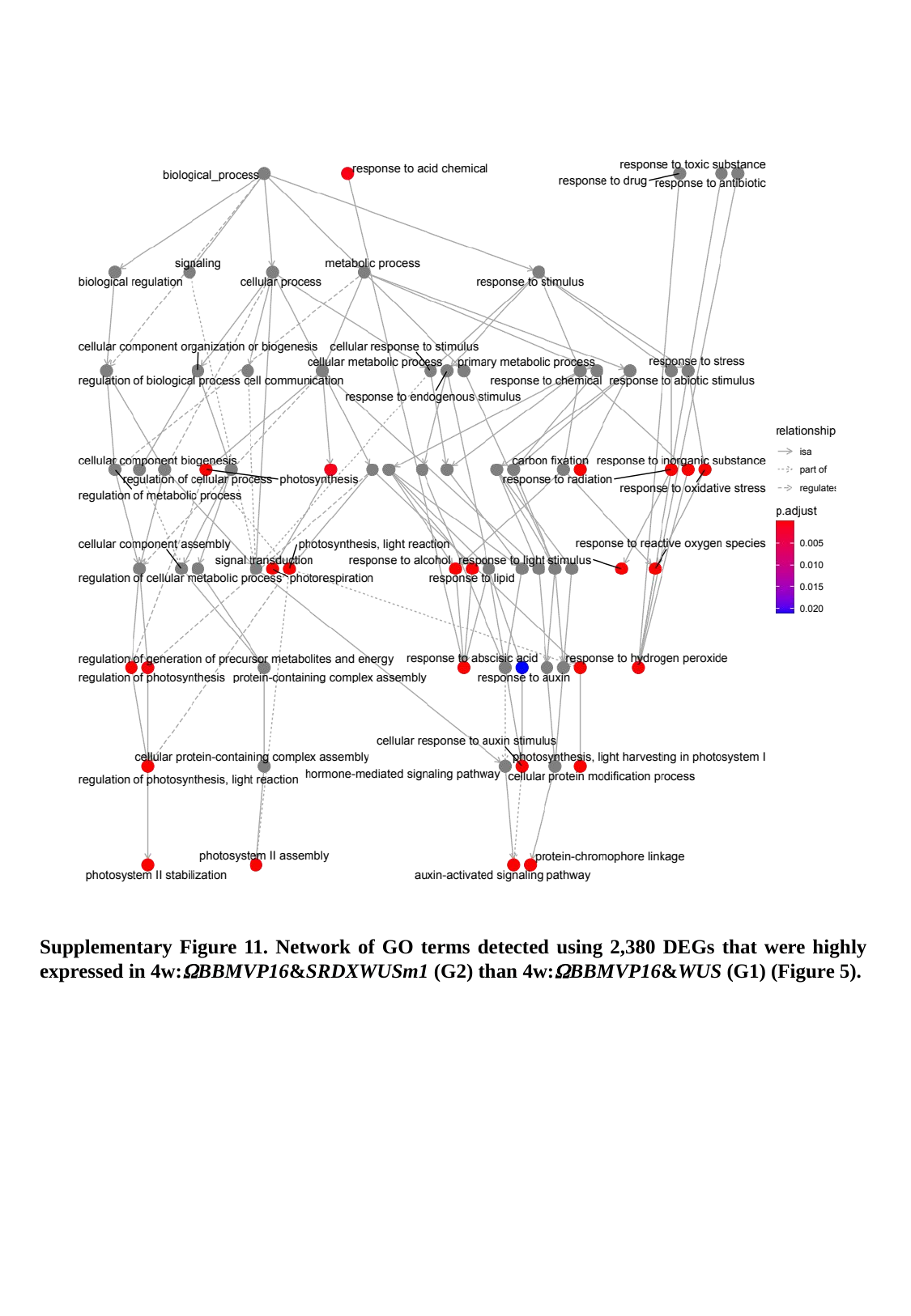

Supplementary Figure 11. Network of GO terms detected using 2,380 DEGs that were highly expressed in 4w:WBBMVP16&SRDXWUSm1 (G2) than 4w:WBBMVP16&WUS (G1) (Figure 5).

## Slide 12
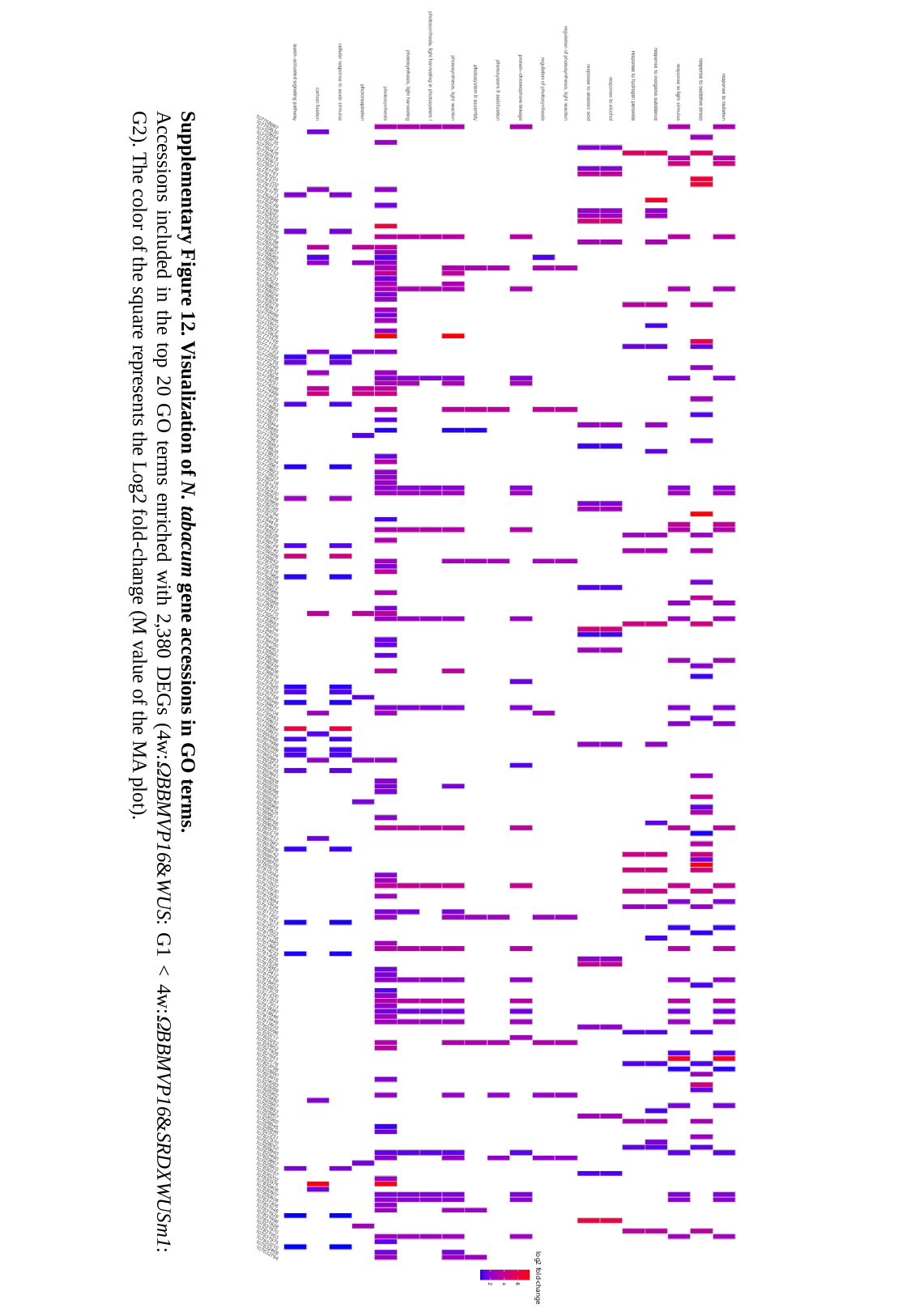

Supplementary Figure 12. Visualization of N. tabacum gene accessions in GO terms.
Accessions included in the top 20 GO terms enriched with 2,380 DEGs (4w:WBBMVP16&WUS: G1 < 4w:WBBMVP16&SRDXWUSm1: G2). The color of the square represents the Log2 fold-change (M value of the MA plot).
log2 fold-change

## Slide 13
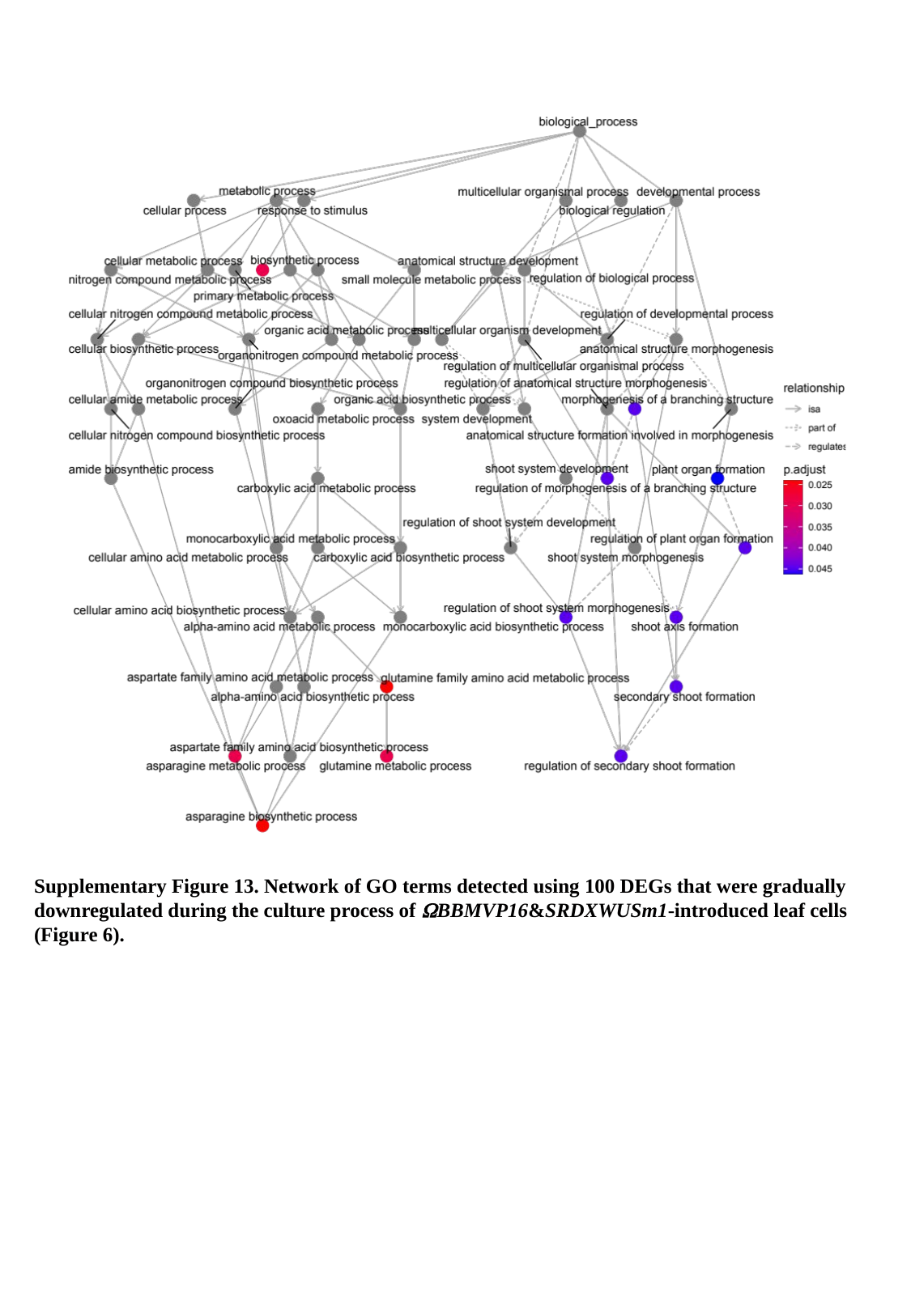

Supplementary Figure 13. Network of GO terms detected using 100 DEGs that were gradually downregulated during the culture process of WBBMVP16&SRDXWUSm1-introduced leaf cells (Figure 6).

## Slide 14
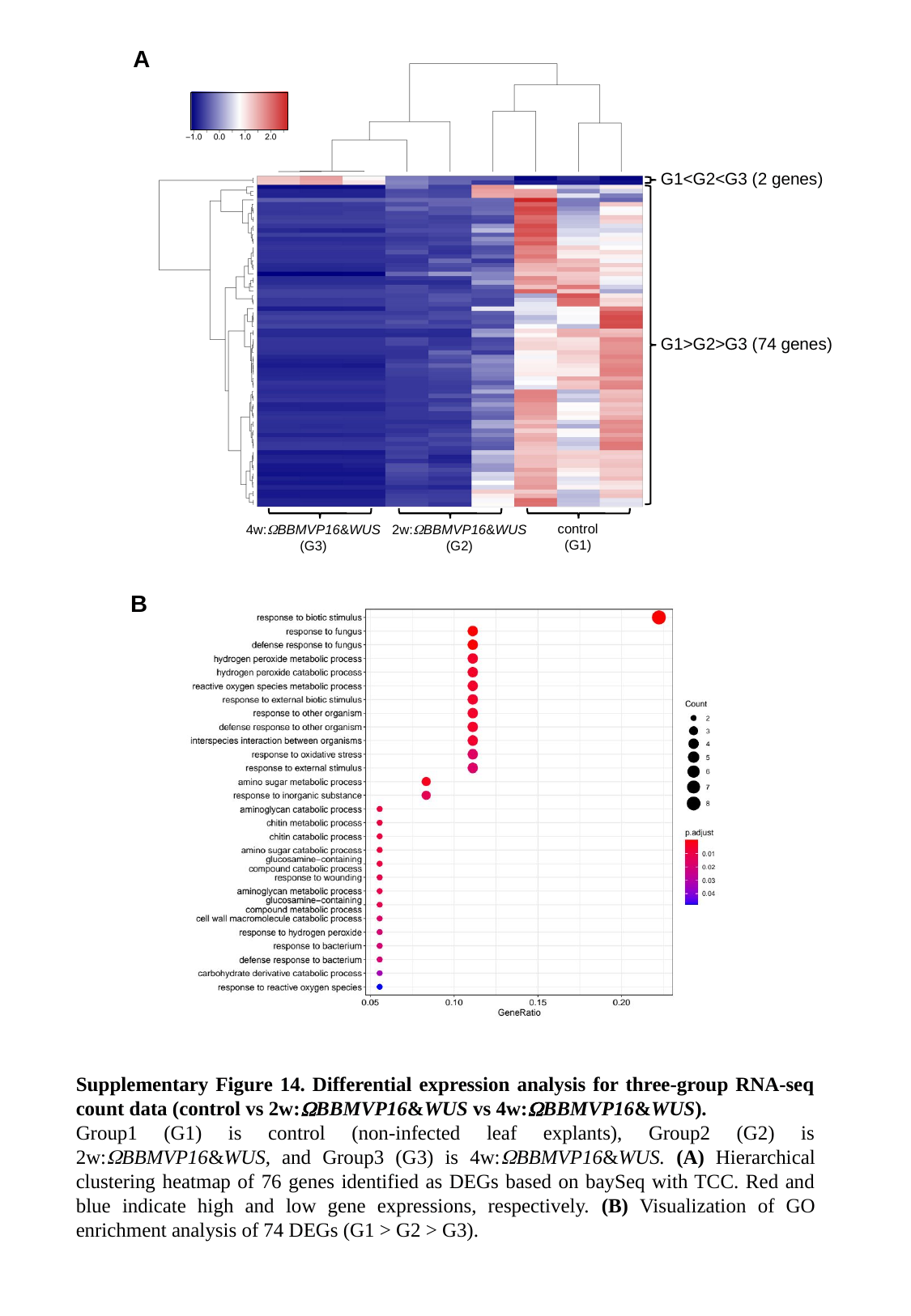

A
G1<G2<G3 (2 genes)
G1>G2>G3 (74 genes)
control
(G1)
2w:ΩBBMVP16&WUS
(G2)
4w:ΩBBMVP16&WUS
(G3)
B
Supplementary Figure 14. Differential expression analysis for three-group RNA-seq count data (control vs 2w:WBBMVP16&WUS vs 4w:WBBMVP16&WUS).
Group1 (G1) is control (non-infected leaf explants), Group2 (G2) is 2w:WBBMVP16&WUS, and Group3 (G3) is 4w:WBBMVP16&WUS. (A) Hierarchical clustering heatmap of 76 genes identified as DEGs based on baySeq with TCC. Red and blue indicate high and low gene expressions, respectively. (B) Visualization of GO enrichment analysis of 74 DEGs (G1 > G2 > G3).

## Slide 15
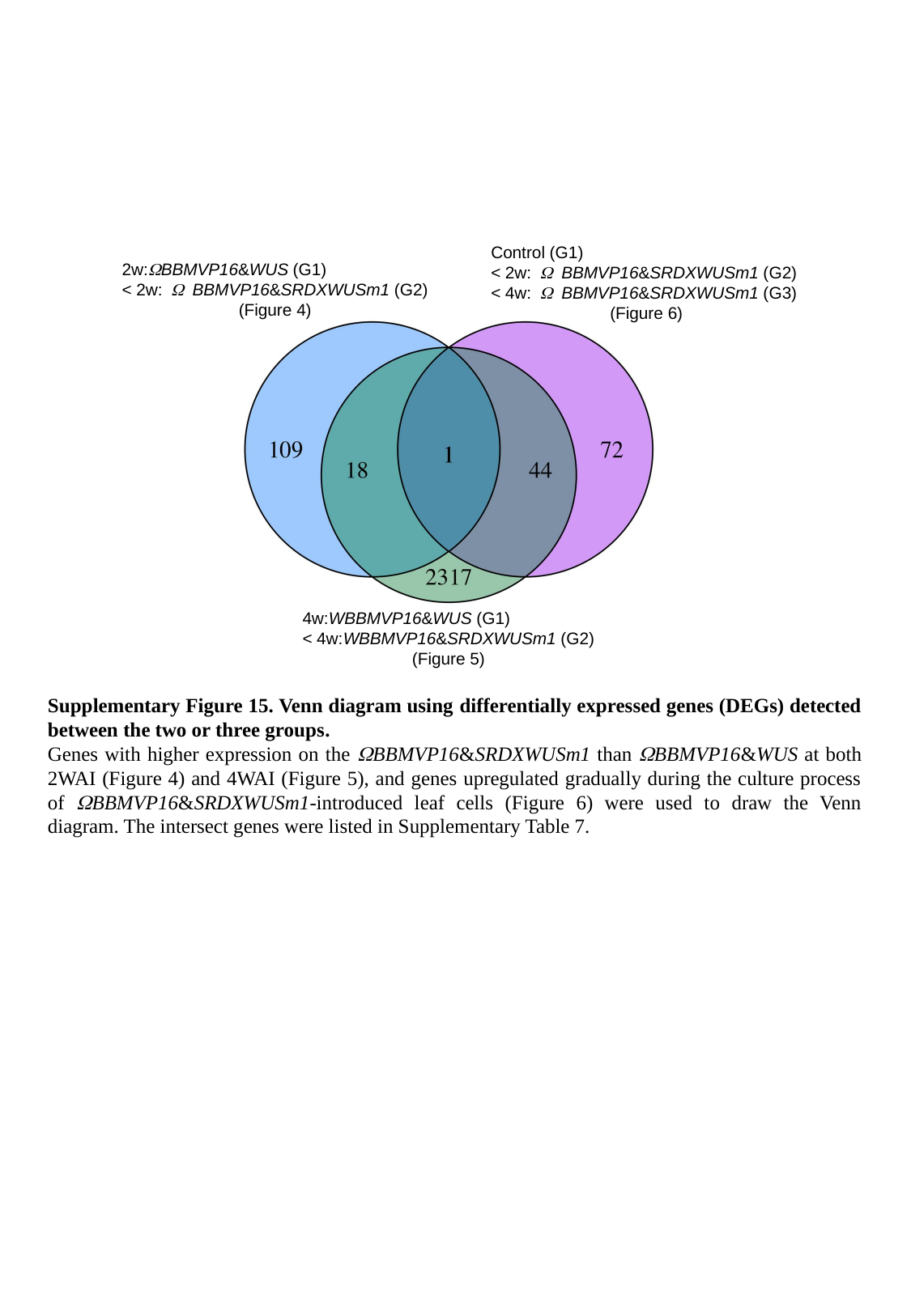

Control (G1)
< 2w: W BBMVP16&SRDXWUSm1 (G2)
< 4w: W BBMVP16&SRDXWUSm1 (G3)
(Figure 6)
2w:WBBMVP16&WUS (G1)
< 2w: W BBMVP16&SRDXWUSm1 (G2)
(Figure 4)
4w:WBBMVP16&WUS (G1)
< 4w:WBBMVP16&SRDXWUSm1 (G2)
(Figure 5)
Supplementary Figure 15. Venn diagram using differentially expressed genes (DEGs) detected between the two or three groups.
Genes with higher expression on the WBBMVP16&SRDXWUSm1 than WBBMVP16&WUS at both 2WAI (Figure 4) and 4WAI (Figure 5), and genes upregulated gradually during the culture process of WBBMVP16&SRDXWUSm1-introduced leaf cells (Figure 6) were used to draw the Venn diagram. The intersect genes were listed in Supplementary Table 7.
