# Supplementary material for: Autonomous differentiation of transgenic cells requiring no external hormone application: the endogenous gene expression and phytohormone behaviors
Source: Front Plant Sci. 2024 Apr 3;15:1308417. doi: 10.3389/fpls.2024.1308417 (PMC11021773; doi:10.3389/fpls.2024.1308417)
Supplement: Supplementary file 1 [file DataSheet_1.zip › Data Sheet 1 (1)/Table S1.DOCX]

**Supplementary Table 1. Primers used in this study.**

| Primer name | Sequence (5’-3’) | | Applications |
| --- | --- | --- | --- |
| AtBBM_CDS_For | ATGAACTCGATGAATAACTGGTTAGGC | | Cloning of *BBM* and genomic PCR |
| AtBBM_CDS_Rev | CTAAGTGTCGTTCCAAACTGAAAACG | | Cloning of *BBM* and genomic PCR |
| BBMseq.(571-594) | GATGTCGTAGAAACTACACCGAAG | | Sequencing |
| infusion WUS F | ATGGAGCCGCCACAGCAT | | Cloning of *WUS* and genomic PCR |
| infusion WUS R | ATCTCGAGTGCGGCCCTAGTTCAGACGTAGCTCAAGAGAAGCGC | | Cloning of *WUS* |
| infusion RPS5A F | AAGGAATTCGCGGCCTTTCAATTTCATGGTGAGGAT | | Cloning of RPS5A promoter |
| infusion RPS5A R | CTGTGGCGGCTCCATTGGCTGTGGTGAGAGAAAC | | Cloning of RPS5A promoter |
| BBM WUS FW | CGGTACCCGGGGATCAATCAAAGGCCATGGAGTC | | Cloning of *BBM* and *WUS* |
| BBM WUS RV | ATCGACCGGCATGCACTAGTTCAGACGTAGCTCA | | Cloning of *BBM* and *WUS* |
| nos terminator FW | TGCATGCCGGTCGATCTA | | Cloning of nos terminator |
| nos terminator RV | CTCCCATATGGTCGAAATTCCCTAGAGTCAAGCAG | | Cloning of nos terminator |
| infusionMYB_F | TCCCGCCTTCAGTTTACAGTCTCAGAAGACCAAAGGGCA | | Cloning of *IbMyb* |
| infusionMYB_R | AAACACTGATAGTTTGATCTAGTAACATAGATGACACCGCGC | | Cloning of *IbMyb* |
| CaMV35S_Asc1 RV | GGCGCGCCTGGTAATTGTGGATCCTCTAGAG | | Sequencing |
| BBM seq1F | ACCACCACTACTACTAACTTCCCC | | Sequencing |
| BBM seq2F | GAGCTTATTGCAGCAACAGCAG | | Sequencing |
| BBM seq1R | AACAGTACCCTCCGGCAACATC | | Sequencing |
| AtBBM_CDS_Rev | CTAAGTGTCGTTCCAAACTGAAAACG | | Cloning of *BBM* |
| M13F | GTAAAACGACGGCCAGT | | Sequencing |
| M13R | GCGGATAACAATTTCACACAGG | Sequencing | |
| Hpa1_RPS5A_inf_F | AATATTCCGTAAGTTAACCGGCTAAAACC | Cloning of RPS5A promoter | |
| RPS5A_inf_Nru1_R | GGATCCTCGGCTGTGGTGAGAGAAACAGAG | Cloning of RPS5A promoter | |
| WUS_inf_F | CACAGCCGAGGATCCACAATTACCAACAACAACAAACAACAA | Cloning of *SRDXWUSm1* | |
| WUS_inf_R_20mer | CTTTGGTCATCTCGAGTGCGGCCGCGAA | Cloning of *SRDXWUSm1* | |
| MinimumCaMV_inf_F | TCGAGATGACCAAAGGGCAATTGAGACTTTTCAAC | Cloning of 35S promoter | |
| MinimumCaMV_inf_F | CATTATGGATCCTCGATCCTCGCGAATTCAAGCTTCCATGG | Cloning of 35S promoter | |
| RT-BBM1 For | GTTGACTCCTCCACCACCAG | RT-PCR | |
| RT-BBM 1Rev | TTCGAGGGTGACACCGAAAG | RT-PCR | |
| RT-WUS1 For | GGAGGATGGGCAAACATGGA | RT-PCR | |
| RT-WUS1 Rev | GAGGAAGCGTACGTCGATGT | RT-PCR | |
| IbMyb_For | ATGGTTATTTCATCTGTATGGTCG | RT-PCR | |
| IbMyb_Rev | TTAACAGTTCTGACAGTAGGTCTA | RT-PCR | |
| WUS_pac1 non_stop | ACGTCGTTAATTAAGTTCAGACGTAGCTCAAGAGAAGCG | genomic PCR | |
| VirD2-F1 | ACCGACCAAACCGCAGCTTA | genomic PCR | |
| VirD2-R1 | CCGCTCGCTCTCCAATGGTA | genomic PCR | |
| IbMyb_seqF1 | GGCCTCGACCTCGGAGATT | genomic PCR | |
| MYB_check_R | ACTCCAACAGATTTGTCCTTCCTCATT | genomic PCR | |
| bbm8-1 | TTCAAACAAGAGGAGGAACAACAAC | qRT-PCR for leaf explant culture | |
| bbm8-2 | CCACAAACGGTAACAGAATCATCAG | qRT-PCR for leaf explant culture | |
| wus6-1 | CGGAAAGATTGAGGGCAAGAA | qRT-PCR for leaf explant culture | |
| wus6-2 | GAGTTGGGTGATGAAGATGGTGT | qRT-PCR for leaf explant culture | |
| NtEF1-qRTfor | TCAGGAGCATGCGTCAAACT | qRT-PCR standard | |
| NtEF1-qRTrev | AGCCTTGGTGACCATAGCAC | qRT-PCR standard | |
| qRT-BBMfor | GTTGACTCCTCCACCACCAG | qRT-PCR for transgenic plant | |
| qRT-BBMrev | TTCGAGGGTGACACCGAAAG | qRT-PCR for transgenic plant | |
| qRT-WUS1_For | GGAGGATGGGCAAACATGGA | qRT-PCR for transgenic plant | |
| qRT-WUS1_Rev | GAGGAAGCGTACGTCGATGT | qRT-PCR for transgenic plant | |
| LOC107795218-1F | GTGGTGAAGCCCCAATTTCAT | qRT-PCR of selected DEG | |
| LOC107795218-1R | AGCAGCCATTGTCCTCCTCT | qRT-PCR of selected DEG | |
| LOC107773826-4F | AAGGCGAAAAACGCCATTGAT | qRT-PCR of selected DEG | |
| LOC107773826-4R | CCATCAGCTGCATCAATTCTGG | qRT-PCR of selected DEG | |
| LOC107801243-2F | TCCACCTCAATCCCAAACACT | qRT-PCR of selected DEG | |
| LOC107801243-2R | CTAACTCCAGGTCTTGGTGGC | qRT-PCR of selected DEG | |
| LOC107782639-3F | TAACAGCGCCCAGAAAAGGA | qRT-PCR of selected DEG | |
| LOC107782639-3R | ACAACCCATTCTGCTCAACGA | qRT-PCR of selected DEG | |
